# Visual deprivation independent shift of ocular dominance induced by cross-modal plasticity

**DOI:** 10.1371/journal.pone.0213616

**Published:** 2019-03-11

**Authors:** Manuel Teichert, Marcel Isstas, Lutz Liebmann, Christian A. Hübner, Franziska Wieske, Christine Winter, Konrad Lehmann, Jürgen Bolz

**Affiliations:** 1 Institute of General Zoology and Animal Physiology, University of Jena, Jena, Germany; 2 Synapses-Circuits-Plasticity, Max Planck Institute of Neurobiology, Martinsried, Germany; 3 Institute of Human Genetics, University Hospital Jena, University of Jena, Jena, Germany; 4 Department of Psychiatry, Technical University Dresden, Dresden, Germany; University of Waterloo, CANADA

## Abstract

There is convincing evidence that the deprivation of one sense can lead to adaptive neuronal changes in spared primary sensory cortices. However, the repercussions of late-onset sensory deprivations on functionality of the remaining sensory cortices are poorly understood. Using repeated intrinsic signal imaging we investigated the effects of whisker or auditory deprivation (WD or AD, respectively) on responsiveness of the binocular primary visual cortex (V1) in fully adult mice. The binocular zone of mice is innervated by both eyes, with the contralateral eye always dominating V1 input over ipsilateral eye input, the normal ocular dominance (OD) ratio. Strikingly, we found that 3 days of WD or AD induced a transient shift of OD, which was mediated by a potentiation of V1 input through the ipsilateral eye. This cross-modal effect was accompanied by strengthening of layer 4 synapses in V1, required visual experience through the ipsilateral eye and was mediated by an increase of the excitation/inhibition ratio in V1. Finally, we demonstrate that both WD and AD induced a long-lasting improvement of visual performance. Our data provide evidence that the deprivation of a non-visual sensory modality cross-modally induces experience dependent V1 plasticity and improves visual behavior, even in adult mice.

## Introduction

It has been demonstrated that the loss or deprivation of one sensory modality can have profound effects on the remaining senses. Such changes are broadly referred to as “cross-modal plasticity” and can improve the functionality of the intact senses [1–6]. Earlier studies suggested that these compensatory enhancements arise because the deprived cortex becomes driven by the spared sensory modalities, broadly referred to as “cross-modal recruitment” [1, 2, 6, 7]. However, there is increasing evidence that functional improvements of the remaining senses can be also attributed to rapid or long-term adaptive changes in the spared sensory cortices. For instance, we could recently show that auditory deprivation (AD) leads to a rapid increase of visually evoked responses in the spared V1 which was accompanied by improvements of V1 spatial frequency and contrast tuning [4, 8]. While these changes appeared most likely due to a rapid disinhibitory effect [4], previous studies demonstrated that more prolonged sensory deprivations lead to plastic alterations in spared primary sensory cortices [6]. For example, a few days of visual deprivation in juvenile mice selectively strengthened layer 4-2/3 synapses in the somatosensory barrel cortex and sharpened the functional whisker-barrel map in layers 2/3 [9]. Similarly, one week of visual deprivation was shown to strengthen thalamo-cortical synapses in the spared primary auditory cortex (A1) of juvenile but also adult mice [10]. These plastic changes were accompanied by increased contrast sensitivity and frequency tuning of A1 neurons [10]. Moreover, in a recent study we could demonstrate that one to two weeks of whisker deprivation (WD) in fully adult mice massively enhanced spatial frequency and contrast tuning of the primary visual cortex (V1) and even markedly improved visually driven behavior [3]. These studies suggest that the ability of sensory cortices to undergo cross-modal plasticity is not restricted to sensory critical periods of early postnatal development, but can also take place in adults, although cortical plasticity levels decline with aging [11–13].

A valuable model of plasticity which typically displays an age dependent decline is the so called OD-plasticity. In young mice, for instance, a monocular deprivation (MD) for a few days shifts the OD away from the closed eye [12, 14]. However, in fully adult mice older than 110 days this type of plasticity is completely absent [11]. In terms of cross-modal plasticity we could recently show that both WD and AD cross-modally restore OD-plasticity in the spared V1 of such fully adult mice [15]. Specifically, combing WD or AD with monocular deprivation (MD) for 7 days induced a shift of the OD in the binocular zone of V1 which was mediated by an increased V1 responsiveness to open eye stimulation [15]. Collectively, these findings suggest that sensory deprivations lead to short-term and, in particular, long-term plastic neuronal alterations, which in turn improve the functionality of spared primary sensory cortices to compensate for the impairment of the deprived sense. However, the time course of events taking place in the spared cortices after the loss or deprivation of another sense is largely unknown. Moreover, the repercussions of late-onset sensory deprivations on functionality of the remaining sensory cortices are still poorly understood.

In order to address these issues we here investigated the cross-modal effects of WD or AD on V1 responsiveness and visually mediated behavior at 0, 3 and 7 days after the deprivation in fully adult mice far beyond their sensory critical periods. For this, we chronically measured V1 responses evoked by visual stimulation of the contralateral and ipsilateral eye using Fourier based periodic intrinsic signal imaging. Strikingly, we found that both WD and AD induced a marked shift of the OD in V1 after 3 days, which was mediated by a strong increase of V1 responses evoked by visual stimulation of the ipsilateral eye. Notably, this OD shift took place without preceding MD, the common paradigm to induce alterations of OD in the visual cortex [12, 14]. Intrinsic imaging after another 4 days of WD or AD revealed that V1 input through the ipsilateral eye and thus also OD completely recovered to baseline levels, suggesting that homeostatic mechanisms readjust activity levels in V1. Finally, we investigated the effects of WD or AD on behavioral visual performance. Strikingly, we found that both spatial frequency and contrast sensitivity thresholds of the optokinetic reflex (OKR) massively improved in a V1 dependent and V1 independent manner. Taken together, our results suggest that the deprivation of a non-visual sensory modality induces plastic changes in the binocular zone of V1 and leads to long-lasting improvements of visual performance, even in fully adult mice.

## Material and methods

### Animals

C57BL/6J (Jackson labs) mice were raised in a group of 2–3 in transparent standard cages (16.5x22.5 cm) on a 12 h light/dark cycle, with food and water available *ad libitum*. Between the chronic experiments each animal was housed alone in a standard cage. The environment in the cage was minimally enriched with cotton rolls and nest material. In our mouse facility the light intensity was about 150–170 lux. As demonstrated recently, these rearing conditions are not sufficient to extend OD plasticity into adulthood [15]. Animal housing in our institution is regularly supervised by veterinaries from the state of Thuringia, Germany. For the present study we used fully adult male and female mice (PD 120–240). All experimental procedures have been performed according to the German Law on the Protection of Animals and the corresponding European Communities Council Directive 2010 (2010/63/EU), and were approved by the Thüringer Landesamt für Lebensmittelsicherheit und Verbraucherschutz (Thuringia State Office for Food Safety and Consumer Protection) under the registration numbers 02-032/16 and 02-050/14. Every effort was made to minimize the number of animals used and their suffering. For or after the experiments stated below, almost all animals were sacrificed by cervical dislocation. Only for following Nissl-staining the mice were perfused transcardially with PBS followed by a 4% PFA (in PBS) solution.

### Optical imaging of intrinsic signals

#### Mouse preparation for optical imaging

To investigate the effects of WD (n = 32) or AD (n = 6) on visually evoked activity of V1 we used Fourier based periodic intrinsic signal imaging [4, 16]. In addition, imaging experiments were performed in n = 9 untreated control mice. As described previously [3, 4], animals were initially anesthetized with 4% isoflurane in a mixture of 1:1 O_2_/N_2_O and placed on a heating blanket for maintaining body temperature (37.5°C). Subsequently, mice received injections of chlorprothixene (20 μg/mouse i.m.) and carprofene (5 mg/kg, s.c.). The animal was fixed in a stereotaxic frame and we removed the skin of the left hemisphere to expose the visual cortex. The exposed area was covered with 2.5% agarose in saline and sealed with a standard microscope glass coverslip. Cortical responses were always recorded through the intact skull. During the experiment isoflurane inhalation anesthesia was applied through a plastic mask and maintained at 0.5–0.6%.

#### Mouse preparation for repeated imaging experiments

Repeated intrinsic imaging in the same mice was performed as described previously [15, 17]. Briefly, after the first imaging session the skin was re-sutured and animals were returned to their standard cages. During the subsequent days animals received a daily injection of carprofen (5 mg/kg, s.c.) for pain prevention. Before the next imaging session (day 3 and 7) the skin was re-opened and imaging was performed as described above.

#### Imaging of visual cortex

Responses of mouse primary visual cortex were recorded described previously [15, 18]. Briefly, the method uses a periodic stimulus that is presented to the animal for some time and cortical responses are extracted by Fourier analysis. In our case, the visual stimulus was a drifting horizontal light bar of 2° width, 100% (or 10%, respectively) contrast and with a temporal frequency of 0.125 Hz. The stimulus was presented on a high refresh rate monitor (Hitachi Accuvue HM 4921-D) placed 25 cm in front of the animal. Visual stimulation was adjusted so that it only appeared in the binocular visual field of the recorded hemisphere (-5° to +15° azimuth, -17° to +60° elevation). The stimulus was presented to the contra or ipsilateral eye or to both eyes for 5 min. Thus, the stimulus was repeated for about 35 times during one presentation period.

#### CCD camera recording procedure

Using a Dalsa 1M30 CCD camera (Dalsa, Waterloo, Canada) with a 135x50 mm tandem lens (Nikon, Inc., Melville, NY), we first recorded images of the surface vascular pattern via illumination with green light (550±2 nm) and, after focusing 600 μm below the pial surface, intrinsic signals were obtained via illumination with red light (610±2 nm). Frames were acquired at a rate of 30 Hz and temporally averaged to 7.5 Hz. The 1024x1024 pixel images were spatially averaged to a 512x512 resolution. We always imaged the left hemisphere of the animals.

#### Data analysis

From the recorded frames the signal was extracted by Fourier analysis at the stimulation frequency and converted into amplitude and phase maps using custom software [18]. In detail, from a pair of the upward and downward maps, a map with absolute retinotopy and an average magnitude map were computed. For data analysis we used the MATLAB standard as described previously [11, 19]. The magnitude component represents the activation intensity of the visual cortex. Since high levels of neuronal activity decrease oxygen levels supplied by hemoglobin and since deoxyhemoglobin absorbs more red light (610±2 nm), the reflected light intensity decreases in active cortical regions. Because the reflectance changes are very small (less than 0.1%) all amplitudes are multiplied with 10^4^ so that they can be presented as small positive numbers. Thus, the obtained values are dimensionless. Amplitude maps were obtained by averaging the response amplitudes of individual pixels from maps to upward and downward moving bars. The ocular dominance index was computed as (C-I)/(C+I) with C and I representing the peak response amplitudes of V1 elicited by contralateral eye and ipsilateral eye stimulation, as described previously [17, 19]. To each condition we took at least three magnitudes of V1 responsiveness and averaged them for data presentation.

### Whisker deprivation (WD) and auditory deprivation (AD)

WD and AD were always performed immediately before the first imaging session or optometry experiments (day 0). WD was performed as described previously [3, 15, 20]. Briefly, animals were deeply anesthetized with 2% isoflurane in a mixture of 1:1 O_2_/N_2_O applied through a plastic mask. The eyes of the animal were protected with silicon oil. Whiskers (macro vibrissae) were plucked bilaterally using fine forceps. Subsequently mice received an injection of carprofene (4 mg/kg, s.c.) for pain prevention and were returned to their standard cages. Over the following days whiskers were re-shaved every other day, and animals received a daily administration of carprofene (4 mg/kg, s.c.).

AD was always induced by bilateral malleus removal as described previously [3, 4, 8]. Briefly, animals were deeply anesthetized with 2% isoflurane in a mixture of 1:1 O_2_/N_2_O applied through a plastic mask. Additionally, mice received a subcutaneous injection of carprofene (4 mg/kg, s.c.) for pain prevention. The eyes of the animal were protected with silicon oil. The tympanic membrane was punctured and the malleus was removed under visual control through this opening using fine sterilized forceps. Great care was taken to avoid any destruction of the stapes and the oval window which is visible through the hearing canal (see [21]). Over the following days animals received a daily administration of carprofene (4 mg/kg, s.c.).

### Monocular deprivation (MD)

We examined whether the cross-modally induced V1 activity changes depend on patterned visual input. For this, in one group of mice (n = 4) we sutured the contra and in another group we sutured the ipsilateral eye (n = 5). MD was always performed after the first imaging session, thus, during the same anesthesia like WD. For this, we increased the isoflurane concentration to 2% in a mixture of 1:1 N_2_O and O_2_. Lid margins of the contra or ipsilateral eye, respectively, were trimmed and an antibiotic ointment was applied. Subsequently the eye was sutured. After MD animals received one injection of carprofene (4 mg/kg, s.c.) and were returned to their standard cages. All animals were checked daily to ensure that the sutured eye remained closed during the MD time. Over the following 3 days animals received a daily administration of carprofene (4 mg/kg, s.c.) for pain prevention.

### Electrophysiology

#### Slice preparation for electrophysiological recordings

350-μm-thick brain slices were prepared from 3-month-old mice (control: n = 4, 3 d WD: n = 3) in preparation aCSF (in mM): 2.5 KCl, 6 MgSO_4_, 1.25 NaH_2_PO_4_, 0.25 CaCl_2_, 260 D-glucose, 25.0 NaHCO_3_, 2 sodium pyruvat, 3 myo inositol, 1 kynurenic acid. At room temperature slices equilibrated for at least 1 h in recording aCSF (in mM): 125 NaCl, 2.5 KCl, 1 MgSO_4_, 1.25 NaH_2_PO_4_, 2 CaCl_2_, 10 D-glucose, 25.0 NaHCO_3_, 2 sodium pyruvat, 3 myo inositol, 0.4 ascorbic acid, gassed with 95% O_2_ / 5% CO_2_, pH7.3.

#### Patch clamp recordings

Coronal brain slices were placed in a submerged recording chamber mounted on an upright microscope (BX51WI, Olympus). Slices were continuously superfused with aCSF (2–3 ml/min, 32 °C, pH 7.3). Patch clamp recordings of miniature excitatory postsynaptic currents (mEPSCs) were performed as described previously [22]. mEPSCs were recorded in layer 4 pyramidal neurons V1. Layer 4 was identified based upon its relatively small cell size and high packing density compared to the surrounding layers. Pyramidal neurons were selected if they displayed a pyramidal-shaped cell body, in agreement with the morphology of principle neurons in the mouse cortex. Inhibitory neurons, which are usually smaller and exhibit very high input resistance values, were avoided [23]. mEPSCs were recorded at a holding potential of −70 mV for at least 5 min in aCSF. Data analysis was performed off-line with the detection threshold levels set to 3 pA for mEPSCs. mEPSCs were isolated by adding tetrodotoxin (0.5 μM, Tocris Bioscience) and bicuculline methiodide (20 μM, Biomol) to block action potential-induced glutamate release and GABA_A_ receptor-mediated mIPSCs, respectively. 30 μM (2*R*)-amino-5-phosphonovaleric acid (dl-APV; Sigma-Aldrich) was added to suppress NMDA currents. The pipette solution contained the following (in mM): 120 CsMeSO_4_, 17.5 CsCl, 10 HEPES, 5 BAPTA, 2 Mg-ATP, 0.5 Na-GTP, 10 QX-314 [*N*-(2,6-dimethylphenylcarbamoylmethyl) triethylammonium bromide], pH 7.3, adjusted with CsOH. The following parameters were determined: frequency and peak amplitude.

### CPP, diazepam and saline injections

To investigate the role of the N-methyl-D-aspartate (NMDA)-receptor on V1 responsiveness and OKR thresholds we administrated the competitive NMDA-receptor blocker (WD+CPP: n = 8) (R,S)-3-(2-carbooxypiperazin-4-yl)propyl-1-phosphonic (CPP, Abcam). CPP was diluted in saline and injected intraperitoneally (i.p.) every 24 h at a dose of 12–15 mg/kg in a volume of 0.12 ml [15, 24]. To increase the level of cortical inhibition in WD mice for imaging experiments we intraperitoneally injected 0.12 ml diazepam solution (in saline, 1mg/kg; n = 4) daily. In control mice (n = 4), we daily injected the same volume of saline (i.p.).

### High performance liquid chromatography (HPLC)

Brain micropunches were taken from 1 mm V1 slices at −3.28 from Bregma from control (n = 5) and WD mice (n = 6) and homogenized by ultrasonication in 20 vol of 0.1 N perchloric acid at 4 °C immediately after collection. A total of 100 ml of the homogenate was added to equal volumes of 1 N sodium hydroxide for measurement of protein content. The remaining homogenate was centrifuged at 17 000 g and 4 °C for 10 min. Glutamate and GABA levels were determined using methods described previously [25]. Briefly, amino acids were precolumn-derivatized with o-phthalaldehyde-2-mercaptoethanol using a refrigerated autoinjector and then separated on a HPLC column (ProntoSil C18 ace-EPS) at a flow rate of 0.6 ml/min and a column temperature of 40 °C. The mobile phase was 50 mM sodium acetate (pH 5.7) in a linear gradient from 5% to 21% acetonitrile. Derivatized amino acids were detected by their fluorescence at 450 nm after excitation at 330 nm.

### Optomotor system

To determine subcortically mediated vision thresholds for spatial frequency and contrast of the optomotor response after WD (n = 11), AD (n = 4) or in untreated control mice (n = 4), we used a virtual optomotor system [26]. Briefly, placed on a platform, freely moving animals were surrounded by moving vertical sine wave gratings of varying spatial frequencies and contrasts. Mice reflexively track grating by head movements (optokinetic reflex, OKR) as long as they can see it [26]. Thresholds for spatial frequencies were measured at 100% contrast and the contrast thresholds were determined at a spatial frequency of 0.2 cycles per degree (cyc/deg). From contrast thresholds contrast sensitivity was calculated (contrast sensitivity = (1/ contrast thresholds)x100)). We measured both spatial frequency and contrast sensitivity for each eye separately and, because they were almost identical, averaged these measurements for data presentation.

### V1 aspiration

To investigate whether V1 is required for the observed effects of WD on the OKR, the monocular and binocular V1 was aspirated bilaterally in WD (n = 3) and control mice (n = 3). First, mice received an injection of carprofene (5 mg/kg) for pain prevention. The correct position of V1 was determined using intrinsic imaging as described previously [4]. Briefly, to localize V1, animals were stimulated with a moving 2° wide horizontal light bar presented on the monitor placed in the right or left visual field at a distance of 25 cm to stimulate right and left eye, respectively. The bar covered 79° azimuth. The obtained retinotopic color coded phase map was then merged with a picture of the skull vascular pattern. Through a burr hole V1 was aspirated bilaterally as described previously [27] and the skin was re-sutured. Animals received subcutaneous carprofene (5 mg/kg) daily for pain prevention.

### Nissl staining

To demonstrate the efficiency of V1 aspiration experiments, we sacrificed mice tested in the Optomotry and performed a Nissl staining in the obtained brain slices. For this, brain sections were fixed in ethanol (95%) containing 5% acetic acid (99.5%) for 30 min. After washing with distilled water sections were incubated in a cresyl violet solution (0.1% in distilled water) for 4 min. After a further incubation in ethanol with ascending concentrations (50%, 70%, 95%, 99.9%) and xylol (98%), sections were embedded in depex (Serva). The sections were observed using a bright field microscope (Olympus) using a 10x objective.

### Experimental design and statistical analysis

To investigate whether WD or AD affect the responsiveness of the visual cortex we performed before-after comparisons of optical imaging data by ANOVA with repeated measures followed by Bonferroni correction. Between-group comparisons were performed by one-way AVOVA, again, followed by Bonferroni correction. Electrophysiological measurements were compared either by a Kolmogorov-Smirnov or unpaired t-test. HPLC were also compared by an unpaired *t-test*. To examine potential effects of the cross-sensory deprivation on the visually mediated OKR, behavioral data of control, WD and AD mice (spatial frequency and contrast thresholds) were first analyzed by a two-way ANOVA with repeated measurements. After this group data were compared by *post hoc* two-tailed unpaired student´s *t*-test. The resulting *p*-values were then Bonferroni corrected. In the graphs, the levels of significance were set as: *p<0.05, **p<0.01, ***p<0.001. Data were analyzed using GRAPHPAD PRISM and SPSS and are presented as means and standard error of the mean (s.e.m) or as measurements of individual animals.

## Results

### Cross-modally induced shifts of ocular dominance (OD)

**Fig 1:**
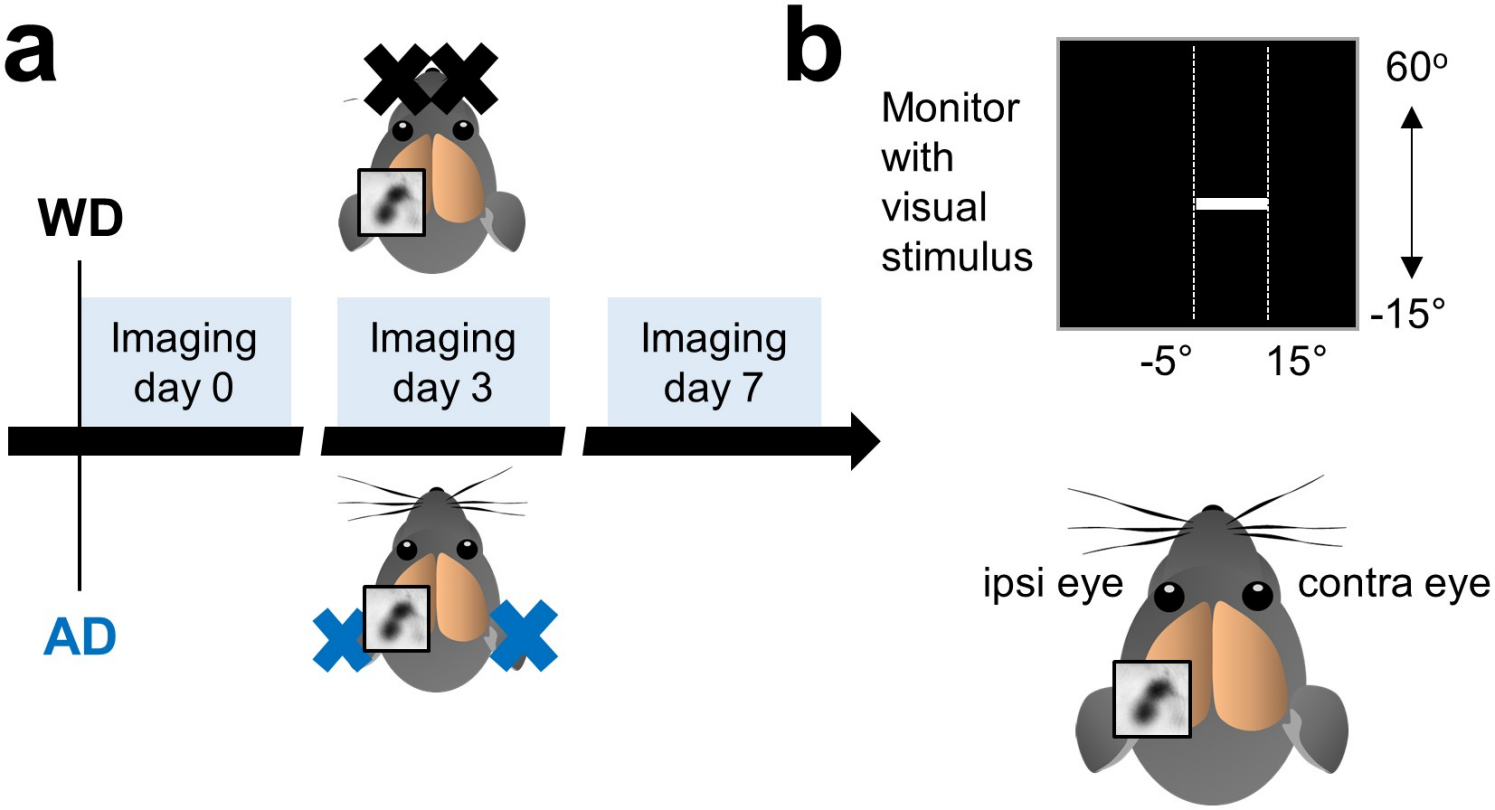
Experimental time course and schematic illustration of intrinsic signal imaging. (**a**) In one group of mice we performed WD by bilaterally plucking all macro-vibrissae and in another group we induced AD by bilateral malleus removal. The first imaging session (day 0) for mapping V1 started immediately after the deprivation followed by a second imaging session at day 3 and a third imaging session at day 7. (**b**) For visual stimulation we used an upward or downward moving white light bar with 100% contrast which was presented in the right binocular visual field. We always recorded V1 responses in the left hemisphere. Thus, according to the position of the recorded hemisphere the left eye represents the ipsilateral and the right eye represents the contralateral eye.

We investigated the cross-modal effects of the deprivation of a non-visual sensory modality on visually evoked activity in the spared binocular V1 in fully adult mice (PD 120–240). For this, we induced either a somatosensory deprivation by bilaterally removing the macro-vibrissae (whisker deprivation, WD; n = 7) [3, 15] or an auditory deprivation (AD, n = 6) by bilateral malleus removal [4, 28] and performed repeated intrinsic signal imaging experiments directly after either WD or AD (day 0) and 3 and 7 days after WD or AD (Fig 1a). Intrinsic signal imaging allows repeated non-invasive measurements of V1 responses evoked by visual stimulation [17, 29] and its reliability has been profoundly validated by electrophysiological recordings [17, 30]. Since the binocular V1 of mice receives input of both eyes, we measured V1 activity evoked by visual stimulation of the contralateral and ipsilateral eye separately (Fig 1b). As a visual stimulus we used a drifting light bar of 100% contrast which was presented in the right binocular visual field (Fig 1b).

**Fig 2:**
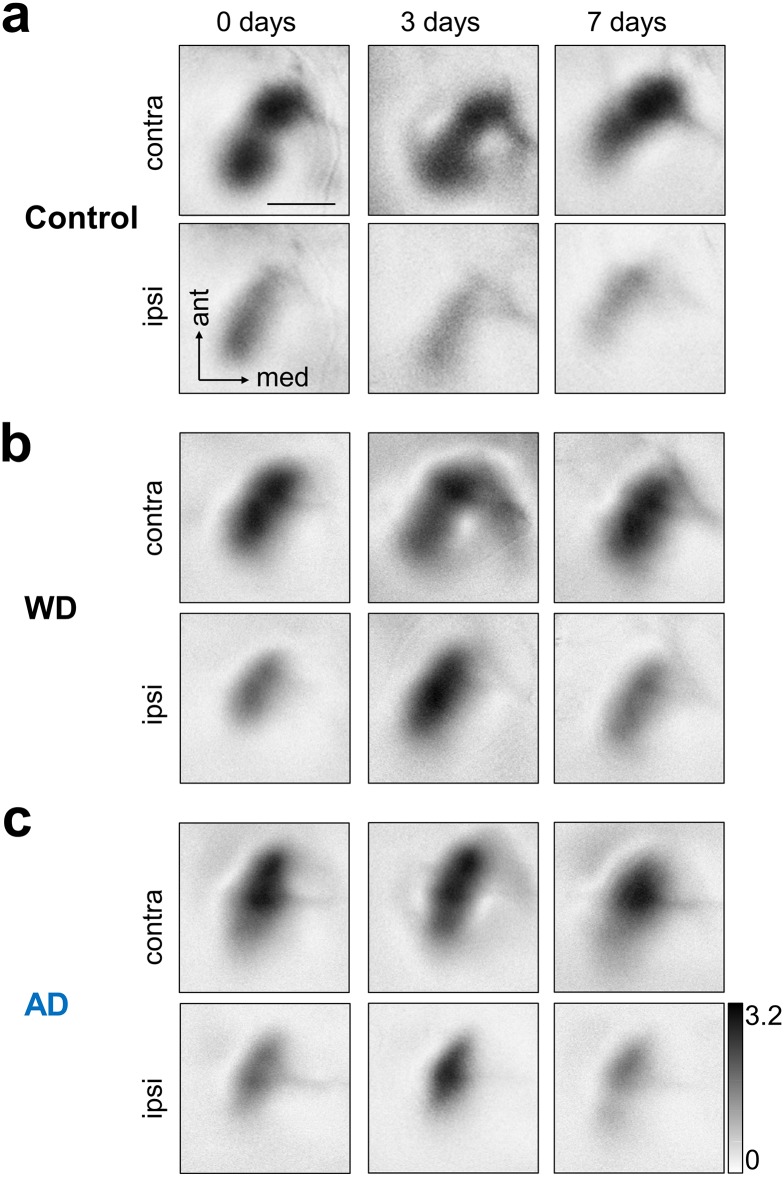
Representative V1 maps evoked by the stimulation of either the contralateral or ipsilateral eye at day 0, 3 and after 7 days. (**a**) Upper row: In normal control mice contralateral eye stimulation always evoked similarly strong V1 responses. Lower row: V1 responses evoked by ipsilateral eye stimulation were equally strong over the time tested but these responses were always weaker that V1 maps obtained after visual stimulation of the contralateral eye. (**b**) Upper row: Like in control mice, WD did not lead to alterations of V1 responses elicited by contralateral eye input. Lower row: However, V1 responses elicited by ipsilateral eye stimulation markedly increased 3 days after WD which was followed by a decrease of V1 response strength 7 days after WD. (**c**) Upper row: Responsiveness of V1 to visual stimulation of contralateral eye remained stable after AD over the time tested. Lower row: 3 days after AD there was a massive increase of V1 responses evoked by ipsilateral eye stimulation which was followed by a recovery of V1 activity 7 days after AD. Thus, the deprivation in both senses (somatosensory or auditory) alters the OD within the binocular zone of V1. Scale bar: 1 mm.

First, we examined whether repeated optical intrinsic imaging *per se* affects visually evoked V1 activity in normal untreated mice (n = 5). Fig 2a depicts representative V1 activity maps evoked by the stimulation of either the contralateral (upper row) or the ipsilateral eye (lower row) obtained at 0, 3 and 7 days. Generally, darker activity maps indicate higher visually driven V1 responses. It is clearly visible that V1 input strength remained unchanged during the time tested, with the contralateral eye always dominating the input to V1, the normal OD ratio for the binocular region of V1 [31]. These results demonstrate that repeated intrinsic imaging provides reliable and stable measurements of sensory evoked V1 activity.

Next, we tested whether WD cross-modally affects responsiveness of V1 to contra- or ipsilateral eye stimulation. As shown in Fig 2b (upper row) V1 activity patches elicited by visual stimulation of the contralateral eye remained equally strong at 0, 3 and 7 days after WD. However, surprisingly, there was a marked increase of V1 responses evoked by ipsilateral eye stimulation 3 days after WD, which was followed by a decrease of V1 input strength back to the level of the V1 maps obtained at 0 days. These results suggest that WD provokes a transient shift of OD within the binocular zone of V1 after 3 days, which during the following 4 days then readjusted to the original level, probably due to homeostatic mechanisms.

We further investigated whether also the deprivation of another non-visual modality, the auditory sense, induces similar cross-modal effects in V1. Fig 2c shows representative V1 maps evoked by the stimulation of either the contralateral (upper row) or ipsilateral eye (lower row) obtained at 0, 3 and 7 days after AD. V1 maps elicited by visual stimulation of the contralateral eye remained stable over the whole time tested. However, like already found after WD, V1 response maps driven by the ipsilateral eye were markedly stronger 3 days after AD. This increase of elicited V1 activity was followed by a decrease back to starting levels 7 days after AD. Thus, our results indicate that the deprivation of non-visual sensory modalities leads to cross-modal alterations of OD. Notably, this took place without monocular deprivation (MD), the common traditional paradigm to induce OD shifts in mammals used up to now since its first description 55 years ago [12, 14, 32].

**Fig 3:**
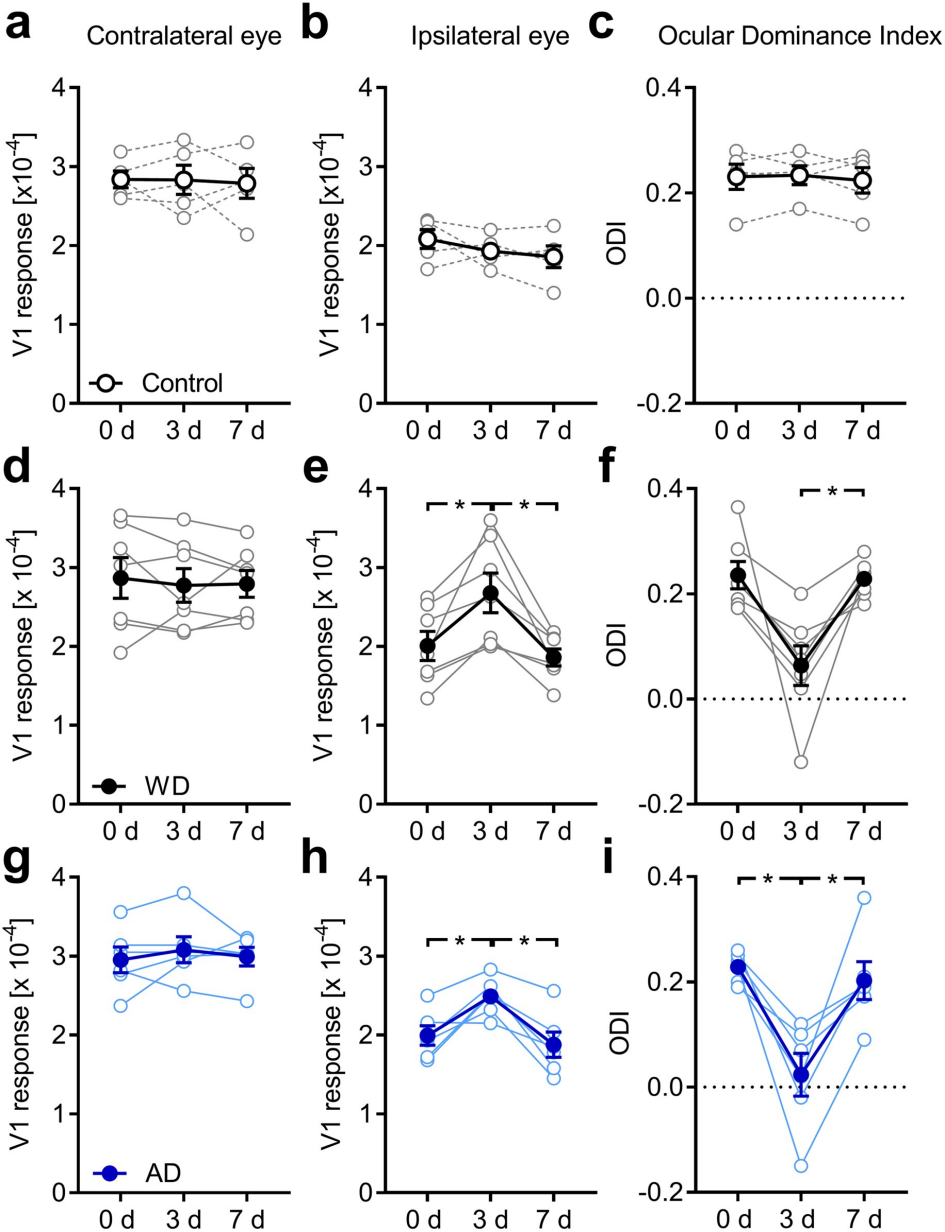
Both WD and AD shift OD in the binocular V1 in fully adult mice. (**a, b**) V1 activity evoked by visual stimulation of the contra or ipsilateral eye in untreated control mice (n = 5) remained unchanged at 0, 3 and 7 days. (**c**) Thus, over the same time course the ODI did not change underlining the reliability of repeated intrinsic signal imaging. (**d**) During one week after WD (n = 7), V1 activity elicited by contralateral eye stimulation was unaltered. (**e**) However, V1 responsiveness to ipsilateral eye stimulation markedly increased 3 days after WD followed by a recovery of V1 activity 7 days after WD. (**f**) These alterations of V1 responsiveness led to an ODI shift towards zero at day 3 which was followed by a readjustment of the ODI 7 days after WD. (**g**) After AD (n = 6) V1 activity elicited by contralateral eye stimulation remained unchanged during the time tested. (**h**) However, V1 responses evoked by visual stimulation of the ipsilateral eye massively increased after 3 days of AD. After the 7 days V1 responses due to the ipsilateral eye input decreased back to the starting level measured at day 0. (**i**) Hence, the ODI displayed a dramatic shift towards zero 3 days after AD which was followed by a complete recovery after one week. Thus, the deprivation of a non-visual input altered OD in the spared V1. Open circles represent measurements of individual animals. Closed circles represent the means of each group ± s.e.m.; *p<0.05, repeated measures AVOVA.

In control animals, neither the cortical response elicited by stimulation of either eye nor, accordingly, the ODI, showed a change over the days of measurement, which was confirmed by ANOVA with repeated measures (factor days across all three variables: F_6,12_ = 1.087, *p* = 0.423, F<1.6 and *p*>0.25 for each single variable, Fig 3a and 3b; Table 1). Thus, these data indicate that intrinsic signal imaging provides stable data of visually evoked V1 activity over the time course examined here.

**Table 1:** The effects of WD and AD on V1 responsiveness and OD. Data are presented as means ± s.e.m.

	0 days	3 days	7 days
***Contra (x10***^***-4***^***)***			
**Control (n = 5)**	2.84 ± 0.11	2.83 ± 0.19	2.79 ± 0.19
**WD (n = 7)**	2.87 ± 0.26	2.77 ± 0.21	2.79 ± 0.17
**AD (n = 6)**	2.95 ± 0.16	3.08 ± 0.17	2.99 ± 0.12
***Ipsi (x10***^***-4***^***)***			
**Control**	2.08 ± 0.12	1.93 ± 0.09	1.86 ± 0.14
**WD**	2.01 ± 0.19	2.68 ± 0.25	1.86 ± 0.11
**AD**	1.99 ± 0.12	2.49 ± 0.10	1.88 ± 0.16
***ODI***			
**Control**	0.23 ± 0.02	0.23 ± 0.02	0.22 ± 0.02
**WD**	0.24 ± 0.03	0.06 ± 0.04	0.23 ± 0.01
**AD**	0.23 ± 0.01	0.02 ± 0.04	0.20 ± 0.04

After WD, however, the measured values changed across days (F_6,20_ = 3.498, *p* = 0.016), which was due to alterations of V1 activity evoked by ipsilateral eye stimulation (F_2,12_ = 11.604, *p* = 0.002) and ODI (F_2,12_ = 11.632, *p* = 0.002), but not contra eye responses (F_2,12_ = 0.3, *p* = 0.746, Fig 3d–3f; Table 1). Pairwise comparisons with Bonferroni correction revealed, that V1 input through the ipsilateral eye significantly increased on day 3 compared to day 0 (*p* = 0.034) and decreased again after 7 days of WD (*p* = 0.021, Fig 3e, Table 1). The corresponding decrease in ODI narrowly missed significance (*p* = 0.056) but was followed by a significant re-increase to starting levels (*p* = 0.016, Fig 3f; Table 1).

After AD, the values also massively changed over the course of the experiment (F_6,16_ = 4.044, *p* = 0.012). In detail, while V1 responsiveness to contralateral eye stimulation remained unaltered (F_2,10_ = 0.402, *p* = 0.679, Fig 3g), V1 activity evoked by visual stimulation of the ipsilateral eye (F_2,10_ = 12.324, *p* = 0.002, Fig 3h) and ODI (F_2,10_ = 13.498, *p* = 0.001, Fig 3i) were found to vary across readings. Pairwise comparisons with Bonferroni correction confirmed that V1 input through the ipsilateral eye increased significantly from day 0 to day 3 (*p* = 0.039) and decreased again after 7 days of AD (*p* = 0.015, Fig 3g, Table 1). As a direct consequence, the ODI followed suit, dropping on day 3 (*p* = 0.016) and rising again on day 7 (*p* = 0.028, Fig 3i, Table 1). These data indicate that both WD and AD lead to an increased V1 activation to visual stimulation after 3 days which is, however, restricted to the ipsilateral eye input. Again, the restoration of V1 activity and ODI after 7 days might be due to homeostatic mechanism adjusting V1 inputs back to baseline levels.

**Fig 4:**
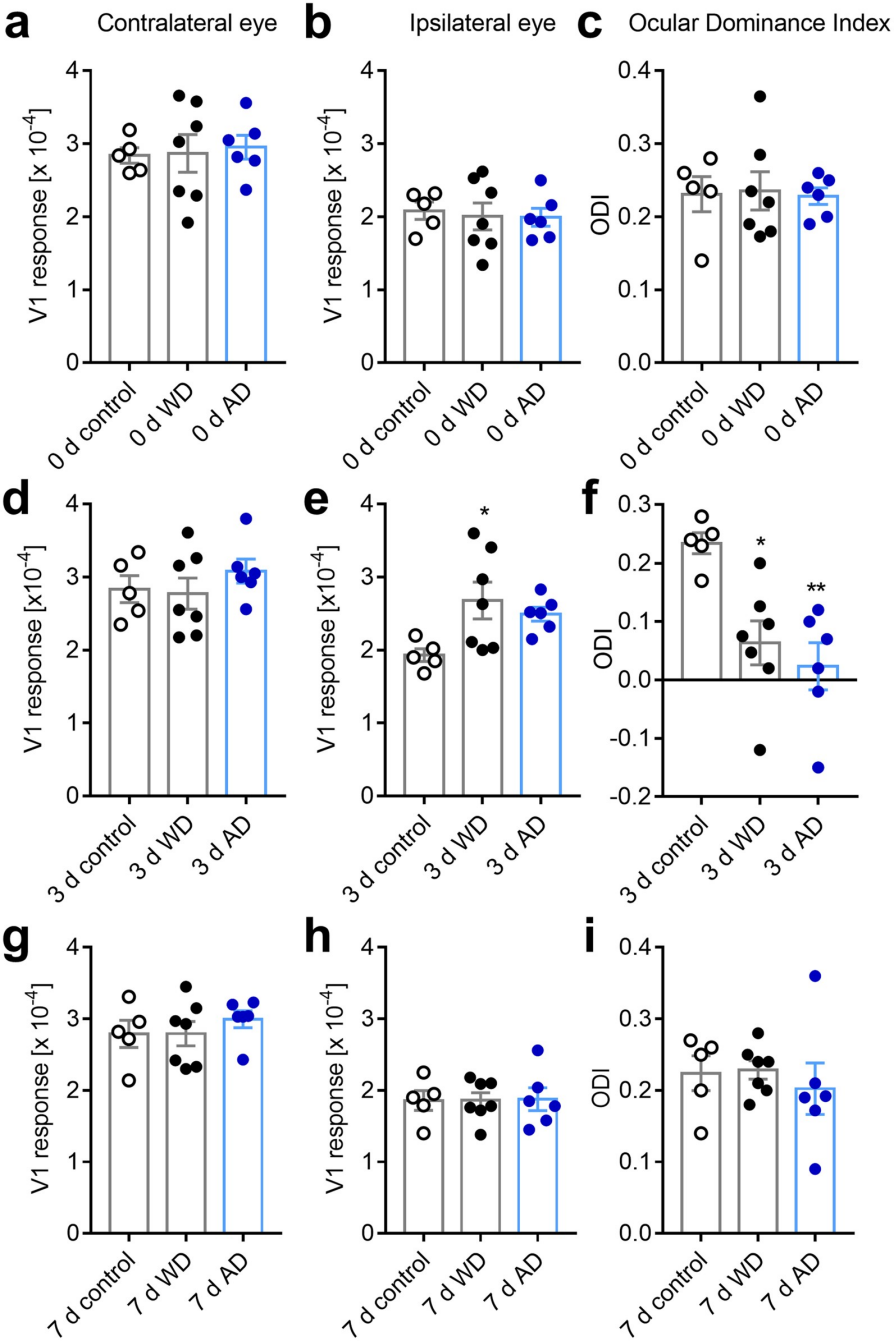
Both WD or AD induce an ODI shift compared to normal control mice. (**a, b**) V1 responses evoked by visual stimulation of either the contra or ipsilateral eye immediately after WD (n = 7) or AD (n = 6) were indistinguishable from values obtained in normal control mice (n = 5). (**c**) Hence, directly after WD or AD there was no alteration of the ODI. (**d**) After 3 days of either WD or AD V1 responses elicited by contralateral eye stimulation was not different from control values. (**d**) However, V1 activity evoked by stimulation of the eye ipsilateral to the recorded hemisphere was increased 3 days after WD or AD compared to control levels. (**f**) Thus, at this time point there was a significant shift of the ODI towards zero. (**g**) After 7 days of WD or AD V1 responses elicited by the stimulation of contralateral eye were unchanged compared to the values of control mice. (**h**) Interestingly, V1 responsiveness to ipsilateral eye stimulation was re-adjusted to control levels after one week of either WD or AD. (**i**) Consequently, the ODI of WD or AD mice was completely restored back to control values after 7 days. Bars represent the means ± s.e.m. and open or filled circles represent measurements of individual animals; *p<0.05, **p<0.01, one way ANOVA.

We also compared our imaging data between the groups. Before the intervention (day 0), all variables were similar in all three groups (contra eye: F_2,15_ = 0.076, *p* = 0.927; ipsi eye: F_2,15_ = 0.089, *p* = 0.916; ODI: F_2,15_ = 0.029, *p* = 0.972, Fig 4a–4c). On the third day after sensory deprivation, however, ANOVA revealed a significant group effect for ipsilateral eye input to V1 (F_2,15_ = 4.096, *p* = 0.038) and ODI (F_2,15_ = 8.726, *p* = 0.003), but not contralateral eye input (F_2,15_ = 0.719, *p* = 0.503, Fig 4d–4f). Pairwise comparison with Bonferroni correction confirmed a significant increase V1 activity evoked by ipsilateral eye stimulation in WD animals (*p* = 0.04), and a significant decrease of the ODI in both AD (*p* = 0.004) and WD (*p* = 0.014) animals compared to control mice. On day 7 after WD or AD, all these differences disappeared again as V1 input through the ipsilateral eye was back to control levels (contra eye: F_2,15_ = 0.518, *p* = 0.606; ipsi eye: F_2,15_ = 0.006, *p* = 0.004; ODI: F_2,15_ = 0.318, *p* = 0.732, Fig 4g–4i).

Taken together, our data indicate that the deprivation of a non-visual sense leads to a selective increase of V1 activity evoked by stimulation of the typically “weaker”, ipsilateral eye and thereby to changes of the OD within the binocular zone of V1. Thus, these results suggest that sensory deprivations of non-visual sensory modalities can cross-modally induce neuronal plasticity in the adult V1.

### Cross modally induced OD changes cannot be explained by a saturation of V1 activity evoked by the contralateral eye

**Fig 5:**
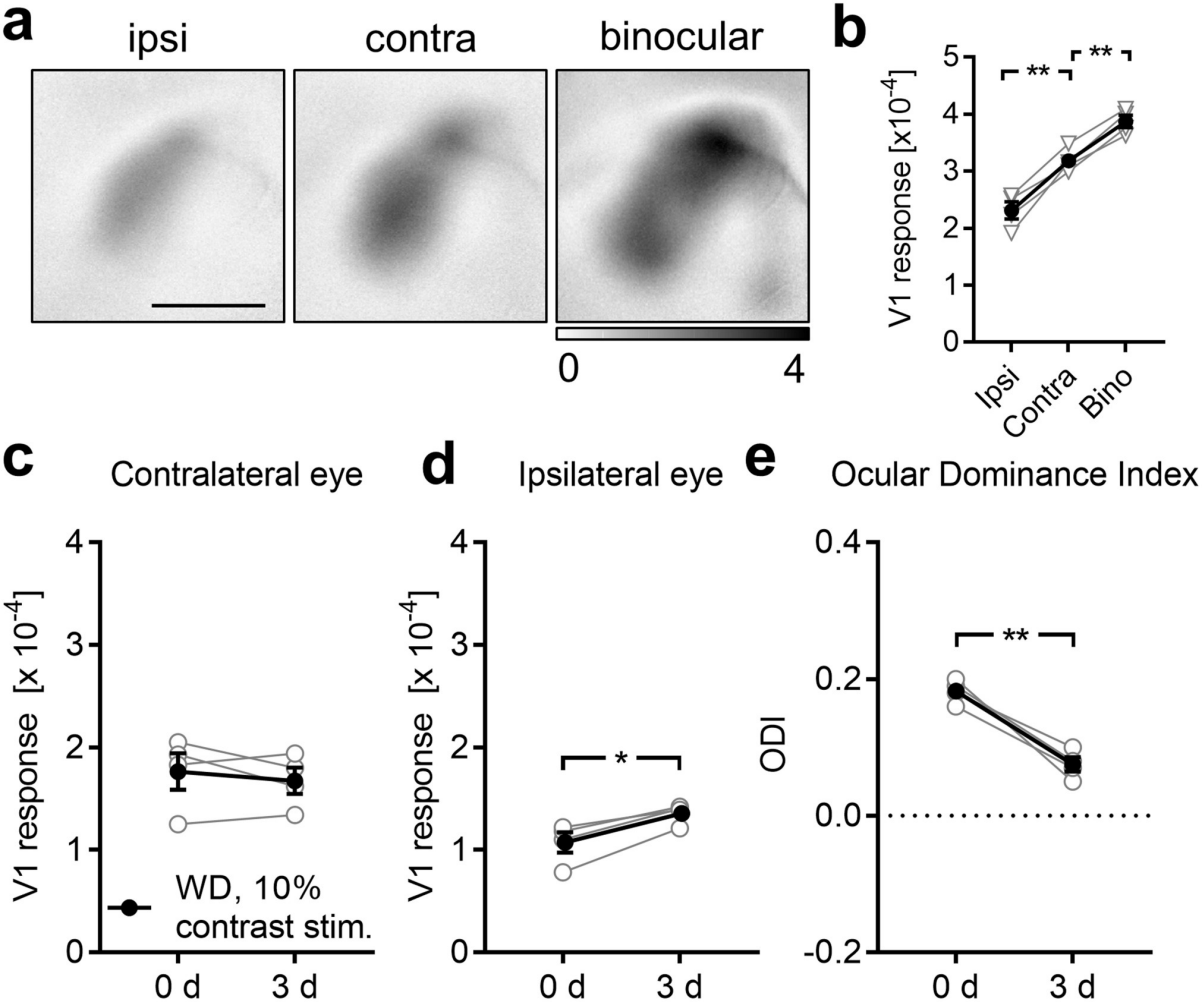
Exclusion of saturation of V1 responses evoked by contralateral eye stimulation. (**a**) Representative V1 amplitude maps evoked by visual stimulation of the ipsi and contralateral eye and elicited by binocular stimulation of normal untreated mice (n = 4). (**b**) V1 responsiveness to ipsilateral eye stimulation was always weaker compared to V1 responsiveness to contralateral eye stimulation. However, V1 activity elicited by visual stimulation of the contralateral eye was significantly weaker than V1 activity evoked by binocular stimulation. Hence, V1 responses, measured with intrinsic signal imaging, are not saturated by the input through the contralateral eye. (**c**) V1 responses evoked by contralateral eye stimulation with a visual stimulus of 10% contrast at 0 and 3 days after WD (n = 4) remained unchanged. (**d**) However, there was a potentiation of V1 responses to the input through the ipsilateral eye between 0 and 3 days after WD. (e) Thus, the ODI significantly shifted towards zero. Hence, visual stimulation with a weaker visual stimulus reveals the same effect of WD on V1 activity like visual stimulation with a strong visual stimulus. Open circles represent measurements of individual animals. Closed circles represent the means of each group ± s.e.m.; *p<0.05, **p<0.01; Scale bar: 1 mm.

So far, we described that both WD and AD lead to a marked ODI shift which was mediated by a selective increase of V1 activity evoked by ipsilateral eye stimulation after 3 days, whereas the contralateral eye input to V1 remains unchanged. Since we were surprised by this unexpected cross-modal effect, we wondered whether the absence of V1 activity changes due to contralateral eye stimulation 3 days after deprivation might be caused by a saturation of the contralateral eye input to V1. To address this issue we first investigated whether V1 activity elicited by contralateral eye stimulation is already saturated in normal control mice (n = 4). For this, we measured V1 responses evoked by monocular ipsilateral and contralateral eye stimulation and also after binocular visual stimulation (Fig 5a). As expected, we found that V1 responses evoked by ipsilateral eye stimulation were always weaker than after stimulation of the contralateral eye (ipsi vs contra: 2.31±0.15 (x10^-4^) vs 3.18±0.11 (x10^-4^), *p* = 0.008; paired *t*-test; Fig 5b). Moreover, V1 activity evoked by contralateral eye stimulation was significantly weaker than after binocular stimulation (contra vs bino: 3.18±0.11 (x10^-4^) vs 3.87±0.11 (x10^-4^), *p* = 0.003; paired *t*-tests; Fig 5b). Thus, these data indicate that V1 responsiveness, as measured by intrinsic signal imaging, is not saturated when evoked by contralateral eye stimulation.

To further exclude a potential saturation effect, we again measured V1 responsiveness to contra and ipsilateral eye stimulation 0 and 3 days after WD (n = 4). However, this time we reduced the contrast of the visual stimulus from 100% to 10%, which generally decreases visually evoked V1 responses [3, 4, 33]. Hence, potential changes of V1 input from the contralateral eye 3 days after WD can be detected. Quantification showed that V1 activity elicited by visual stimulation of the contralateral eye still remained unchanged between 0 and 3 days after WD (0 d vs 3 d: 1.77±0.18 (x10^-4^) vs 1.68±0.13 (x10^-4^), *p* = 0.47; paired *t*-test; Fig 5c) whereas ipsilateral eye input to V1 significantly increased again (0 d vs 3 d: 1.07±0.10 (x10^-4^) vs 1.36±0.05 (x10^-4^), *p* = 0.01; paired *t*-test; Fig 5d). Thus, the differential V1 activity changes caused a marked reduction of the ODI 3 days after WD (0 d vs 3 d: 0.18±0.009 vs 0.08±0.01, *p* = 0.006; paired *t*-test; Fig 5e). These data suggest that cross-modally induced OD changes in V1 are independent of the strength of the visual stimulus. Thus, the absence of V1 activity changes evoked by contralateral eye stimulation 3 days after WD, described above, is not caused by a saturation of the contralateral eye input to V1.

### Cross-modally induced changes of OD require visual experience exclusively through the ipsilateral eye

**Fig 6:**
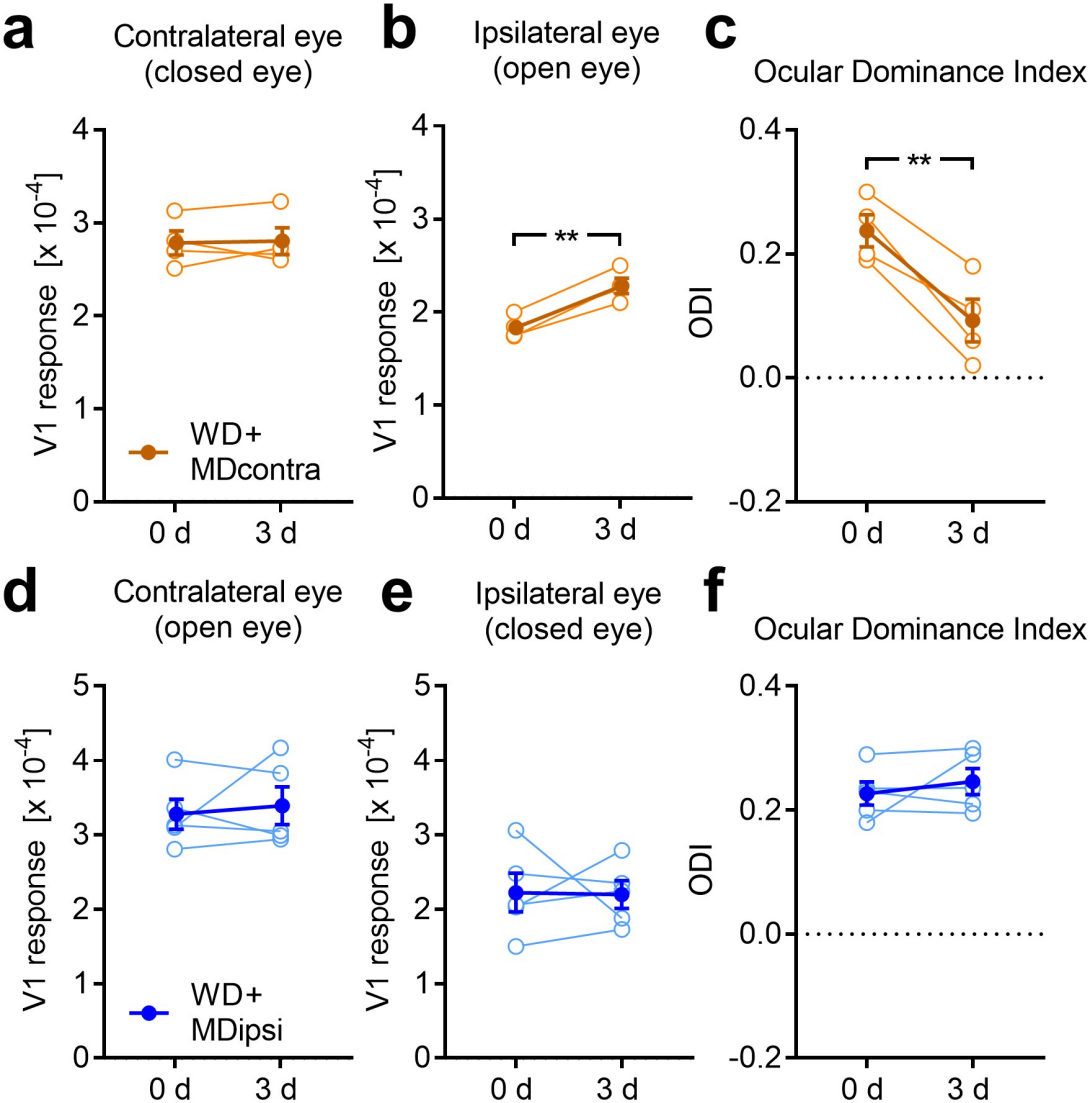
Cross-modally induced ODI shift requires patterned vision through the ipsilateral eye. (**a**) Combining WD with a MD of the contralateral eye (n = 4) did not lead to changes of V1 responses evoked by the contralateral eye between 0 and 3 days after WD. (**b**) However, there was a significant increase of V1 activity elicited by visual stimulation of the ipsilateral eye, like found after WD only. (**c**) Thus, the ODI markedly shifted towards zero. (**d, e**) In contrast, if we combined WD with a MD of the ipsilateral eye (n = 5), V1 activity evoked by both contra and ipsilateral eye stimulation remained statistically unchanged after 3 days. (**f**) Moreover, there was no ODI shift after this treatment suggesting that patterned vision through the ipsilateral eye is required for cross-modally induced OD shifts. Open circles represent measurements of individual animals. Closed circles represent the means of each group ± s.e.m.; *p<0.05, **p<0.01.

It has been suggested that plastic changes in a spared primary sensory cortex require sensory experience through its main input [10]. Thus, we next asked whether patterned visual input through the contra or ipsilateral eye is necessary to provoke V1 activity changes 3 after WD. To address this question we first combined WD with MD of the contralateral eye (n = 4) and measured V1 responsiveness at 0 and 3 days using intrinsic signal imaging. We found that V1 responses evoked by visual stimulation of the contralateral (closed) eye remained unchanged whereas V1 activity driven by the ipsilateral (open) eye input was still increased 3 days after WD and MD (contra: 0 d vs 3 d: 2.79±0.13 (x10^-4^) vs 2.81±0.14 (x10^-4^), *p* = 0.86; ipsi: 0 d vs 3 d: 1.83±0.06 (x10^-4^) vs 2.30±0.08 (x10^-4^), *p* = 0.002; paired *t*-tests; Fig 6a and 6b). This again led to a significant reduction of the ODI (0 d vs 3 d: 0.24±0.03 vs 0.09±0.03, *p* = 0.009; paired *t*-test; Fig 6c), similar to WD mice with open contralateral eyes. Moreover, the percentage increase of V1 activity evoked by ipsilateral eye stimulation was statistically indistinguishable from WD mice without MD described in the first paragraph of the results (WD+MD (contra): 24.58%±2.38%; WD only: 35.71%±12.38%, *p* = 0.46; unpaired t-test). Thus, our data suggest that cross-modally induced changes of OD do not require patterned visual input through the contralateral eye.

Next, we investigated whether experience of patterned vision through the ipsilateral eye is required for WD induced changes of OD. Combining WD with a MD of the ipsilateral eye (n = 5) did not lead to changes of the contralateral eye input to V1 (0 d vs 3 d: 3.28±0.20 (x10^-4^) vs 3.40±0.25 (x10^-4^), *p* = 0.67; paired *t*-test; Fig 6d). However, this treatment abolished the increase of V1 responsiveness to ipsilateral eye stimulation after 3 days of WD (0 d vs 3 d: 2.23±0.26 (x10^-4^) vs 2.20±0.19 (x10^-4^), *p* = 0.94; paired *t*-test; Fig 6e). Hence, the ODI did not change after these interventions (0 d vs 3 d: 0.23±0.02 vs 0.25±0.02, *p* = 0.45; paired *t*-test; Fig 6f). These data suggest that cross-modal changes of V1 activity after WD exclusively depend on visual experience through the eye ipsilateral to the recorded hemisphere.

### WD cross-modally increases mEPSC amplitudes in layer 4 of V1

**Fig 7:**
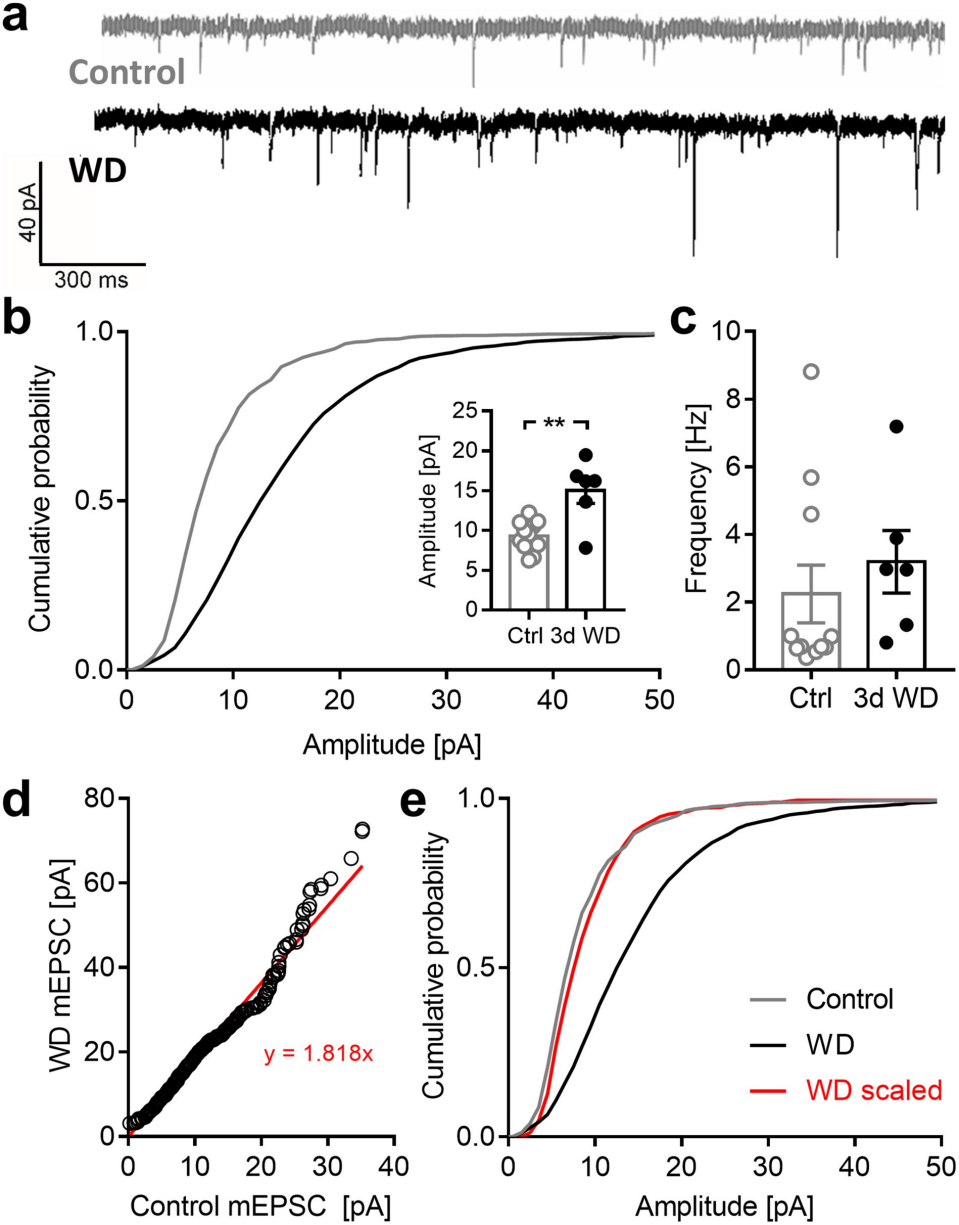
WD cross-modally increases mEPSC amplitudes in V1 layer 4. (**a**) Representative traces of mEPSCs recorded in control mice (n = 4) and 3 d after WD (n = 3) (**b**) Cumulative distribution of all mEPSC amplitudes was right shifted in WD mice compared to control animals. Hence, the mean amplitude of mEPSCs was significantly increased in WD mice. These results suggest that WD induces synaptic plasticity in V1 layer 4. (**c**) Mean frequency of mEPSCs was unaltered after WD. (**d**) Plot of rank ordered mEPSC amplitudes from control and WD mice. The red line represents a linear regression of the data points. (**e**) Cumulative histograms of mEPSC amplitudes. Individual mEPSC amplitudes of WD mice were transformed with the equation y = 1.818x. The distribution of the transformed values is significantly different with the distribution of control values (Kolmogorov-Smirnov test). Bars represent means together with s.e.m., Open and filled circles represent measurements of individual animals; **p<0.01.

It has been demonstrated that a sensory deprivation can re-induce synaptic plasticity of thalamo-cortical layer 4 synapses in a spared sensory cortex in adult mice [10]. Hence, to get insights into potential mechanisms that may underlie the cross-modal cortical changes described above, we next examined the effects of WD on the strength of layer 4 synapses of pyramidal cells in the spared V1. For this, we performed whole-cell recordings in acute V1 slices of normal control mice (11 cells, n = 4 mice) and animals 3 d after WD (6 cells, n = 3 mice). Fig 7a depicts a rpresentative mEPSC trace of a control cell and a WD cell. It is clearly visible that mEPSC amplitudes were increased after 3 days of WD. The cumulative distribution of mEPSC amplitudes of all cells examined was markedly shifted to the right, towards higher amplitudes (*p* = 0.0008; Kolmogorov-Smirnov test; Fig 7b). Hence, on average, there was a significant increase in α-amino-3-hydroxy-5-methyl-4-isoxazolepropionic acid receptor (AMPAR) mediated mEPSC amplitudes (control: 9.31±0.58 (pA), 3 d WD: 15.02±1.63 (pA), *p* = 0.0011; unpaired *t*-test; Fig 7b) suggesting a strengthening of excitatory synapses. However, we did not find changes in frequency of miniature excitatory postsynaptic currents (mEPSC) after WD (control: 2.42±0.85 (Hz), 3 d WD: 3.19±0.93 (Hz), *p* = 0.49; unpaired *t*-test; Fig 7c). Is has been described that changes in the distribution of mEPSC amplitudes can be either multiplicative, if the strength of all neurons excitatory synapses is changed by the same factor (synaptic scaling), or non-multiplicative, if the synaptic changes are not uniform across the sampled synapses [28, 34–36]. Hence, we used the standard method to examine in which manner WD cross-modally affected layer 4 synapses in V1: first, rank ordered mEPSC amplitudes of WD mice were plotted against rank-ordered mEPSC amplitudes of control animals. This plot was fitted by a linear regression revealing the scaling function, y = 1.818x (Fig 7d), as described previously [34, 37]. Then, we transformed individual mEPSC amplitudes of WD mice with this equation and constructed a cumulative plot (WD scaled, Fig 7e). The resulting distribution of the scaled WD data was significantly different from the distribution of control data (*p* = 0.007, Kolmogorov-Smirnov test; Fig 7e) suggesting that only a subset of synapses in layer 4 pyramidal cells was strengthened after WD.

In conclusion, our data indicate that WD cross-modally increases the strength of excitatory V1 layer 4 synapses. These may include thalamo-cortical synapses driven by the ipsilateral eye. Typically, strengthening of thalamo-cortical synapses leads to an increased sensory driven responsiveness of primary sensory cortices [10, 38]. Consistent with these observations is our finding that V1 responses evoked by ipsilateral eye stimulation were increased 3 d after WD, as revealed by intrinsic imaging. Taken together, these data suggest that WD cross-modally re-induces synaptic plasticity in the spared V1.

### WD cross-modally increases the E/I ratio in V1

**Fig 8:**
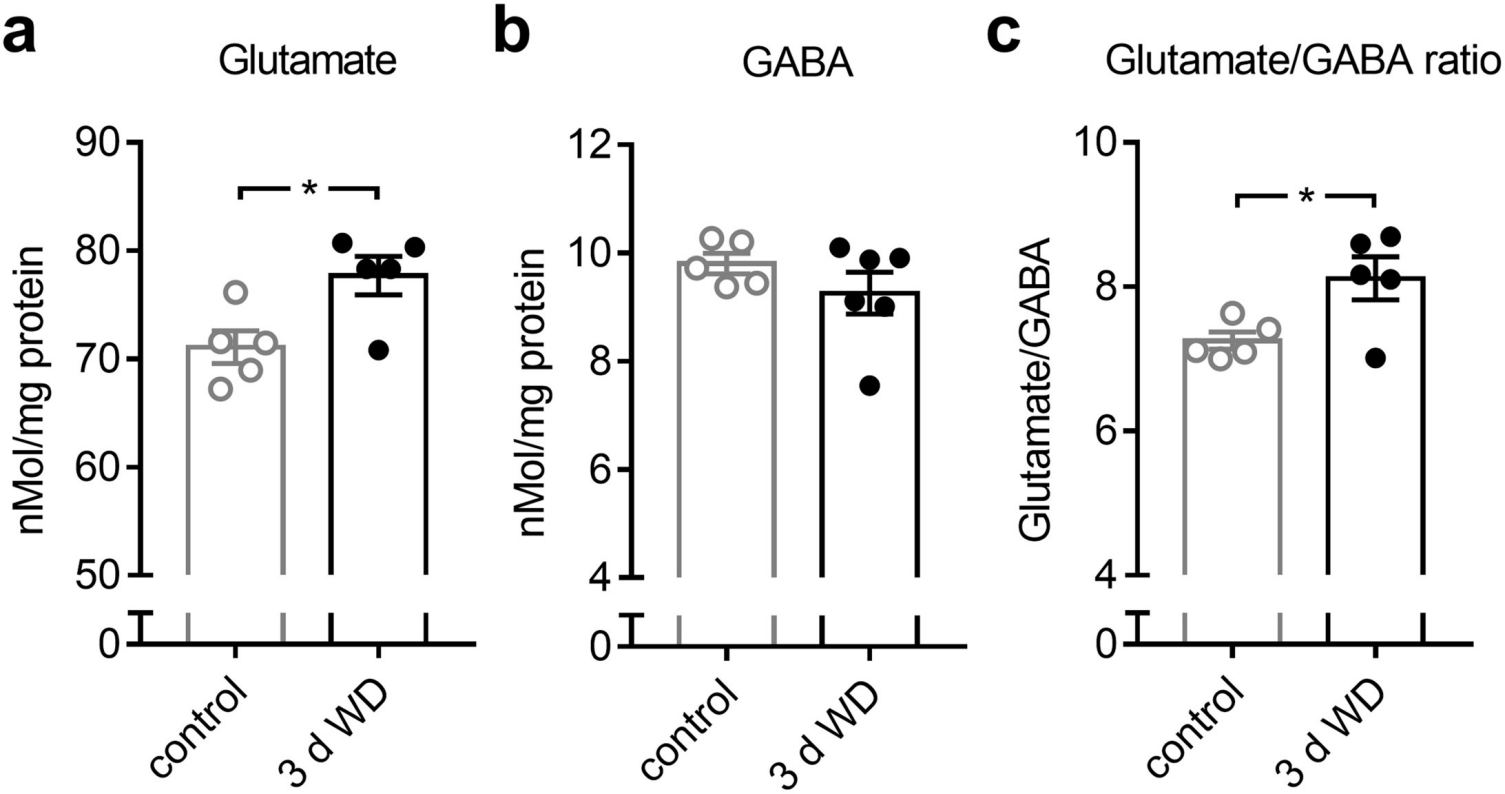
Concentration changes of neurotransmitters in V1 after WD revealed by post-mortem HPLC analyzes. (**a**) Compared to the V1 glutamate level of control mice (n = 5), there was a significant increase of V1 glutamate content 3 days after WD (n = 5). (**b**) There was slight but not significant reduction of the V1 GABA concentration at 3 days after WD (n = 6) compared to controls. (**c**) The glutamate/GABA ratio was markedly increased at 3 after WD. Bars represent means together with s.e.m., Open and filled circles represent measurements of individual animals; *p<0.05.

Experience dependent V1 plasticity after MD that leads to changes in OD, typically declines with aging and is completely absent in fully adult mice [11], as used in the present study. However, previous studies could demonstrate that increasing the cortical excitation/inhibition (E/I) ratio is a central hub for the restoration of visual plasticity in the adult V1 [39–42]. Hence, as a next step, we examined whether WD for 3 days leads to cross-modal changes in V1 glutamate and GABA levels. For this, we quantified levels of glutamate and GABA by post-mortem HPLC analyzes of V1 tissue from control mice (n = 5) and WD mice 3 days after WD (n = 6). Quantification showed that there was a significant increase in V1 glutamate levels 3 days after WD (control vs 3 d WD: 71.07±1.5 (nMol/mg protein) vs 77.70±1.79 (nMol/mg protein), *p* = 0.02; unpaired *t*-test; Fig 8a). Moreover, V1 GABA content slightly decreased by about 6% after WD, which was, however, not statistically significant (control vs 3 d WD: 9.80±0.19 (nMol/mg protein) vs 9.26±0.39 (nMol/mg protein), *p* = 0.27; unpaired *t*-test; Fig 8b). Due to the differential regulations of glutamate and GABA levels in V1 after WD, the glutamate/GABA ratio significantly increased 3 days after WD (control vs 3 d WD: 7.25±0.12 vs 8.11±0.30, *p* = 0.02; Fig 8c). These results suggest that WD cross-modally alters the E/I balance in V1 in favor of excitation, which might in turn set the adult V1 back into a plastic stage.

### Cross-modal changes of V1 activity depend on V1 GABA levels and NMDA receptor activation

**Fig 9:**
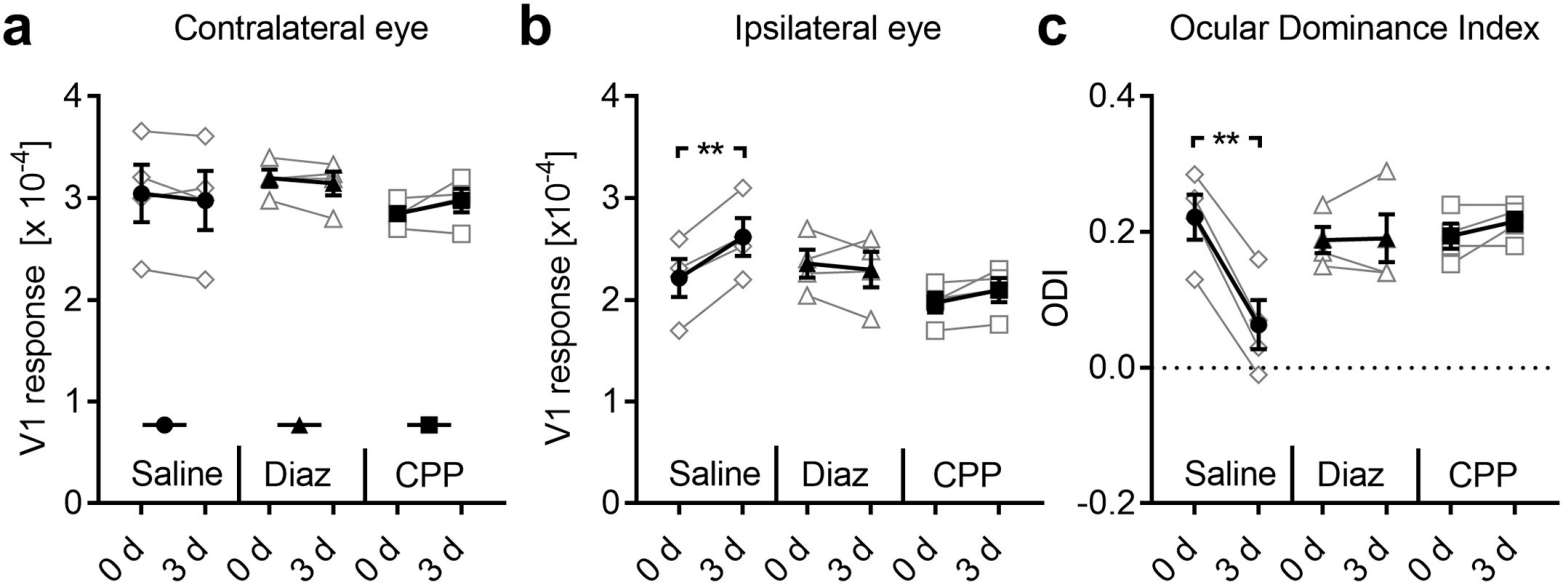
Both increasing inhibition and blocking NMDA receptor activation block cross-modally induced V1 plasticity. (**a**) In WD mice treated with saline (n = 4) or diazepam (n = 4) or CPP (n = 4) V1 responses evoked by visual stimulation of the contralateral eye remained unchanged. (**b**) There was a significant increase of V1 responses elicited by ipsilateral eye stimulation in WD+Saline mice. However, these changes were completely abolished by diazepam or CPP injections. (**c**) We found a significant reduction of ODI in saline treated WD mice, whereas ODI did not change after diazepam of CPP administration. Taken together, our data suggest that cross-modally induced alterations of V1 OD depend on increased glutamateric excitation and NMDA receptor activation. Open circles represent measurements of individual animals. Closed circles represent the means of each group ± s.e.m.; **p<0.01.

We next investigated whether the increase of the V1 glutamate/GABA ratio after WD was related to cross-modally induced V1 activity changes. Hence, to compensate for the increase of glutamatergic excitation, we artificially raised cortical GABAergic inhibition by daily systemic administration of diazepam in WD mice (n = 4) and measured V1 responsiveness at 0 and 3 days using intrinsic imaging. Diazepam, administrated systemically or locally, is a common tool for enhancing cortical inhibition, since it increases GABA receptor mediated currents [43–45]. In control animals (n = 4) we also performed WD but administrated saline systemically. As expected, saline treatment did not influence the cross-modal effects of WD on V1, as V1 activity evoked by visual stimulation of the contralateral eye was unchanged during the time tested, whereas the ipsilateral eye input significantly increased at day 3 after WD (contra: 0 d vs 3 d: *p* = 0.38; ipsi: 0 d vs 3 d: *p* = 0.006; paired *t*-tests; Fig 9a and 9b; Table 2). Consequently, ODI significantly decreased after WD (0 d vs 3 d: *p* = 0.002; paired *t*-tests; Fig 9c; Table 2). However, in WD animals treated with diazepam, both the contralateral and ipsilateral eye input strength in V1 and thereby the ODI also remained unchanged at 3 days (contra: 0 d vs 3 d: *p* = 0.36; ipsi: 0 d vs 3 d: *p* = 0.61; ODI: 0 d vs 3 d: *p* = 0.89; paired *t*-tests; Fig 9a, 9b and 9c; Table 2). Thus, increasing cortical inhibition abolished the WD induced activity changes in V1. These results suggest that the WD induced increase of the V1 E/I ratio is causal for the OD shift observed 3 days after WD.

**Table 2:** The effects of diazepam and CPP administration on cross-modally induced V1 activity changes after WD. Data are presented as means ± s.e.m.

	0 days	3 days
***Contra (x10***^***-4***^***)***		
**WD+Saline (n = 4)**	3.04 ± 0.28	2.97 ± 0.29
**WD+Diaz (n = 4)**	3.19 ± 0.09	3.14 ± 0.12
**WD+CPP (n = 4)**	2.85 ± 0.06	2.97 ± 0.12
***Ipsi (x10***^***-4***^***)***		
**WD+Saline**	2.21 ± 0.19	2.61 ± 0.19
**WD+Diaz**	2.35 ± 0.14	2.30 ± 0.17
**WD+CPP**	2.00 ± 0.10	2.09 ± 0.12
***ODI***		
**WD+Saline**	0.22 ± 0.03	0.06 ± 0.04
**WD+Diaz**	0.19 ± 0.02	0.19 ± 0.04
**WD+CPP**	0.19 ± 0.02	0.22 ± 0.01

Next, we tested the hypothesis that WD induced V1 activity changes might rely on NMDA receptor (NMDAR) activation. Previous investigations could demonstrate an involvement of NMDARs in experience dependent V1 plasticity, as systemic administration of the competitive NMDA receptor antagonist CPP or genetic deletion of cortical NMDARs abolished plastic alterations in V1 [15, 24, 46]. Moreover, we could recently show that blocking NMDAR activation by systemic administration of CPP abolished cross-modally induced restoration of ocular dominance plasticity after 7 days of monocular deprivation [15]. Hence, here, WD mice received daily injections of CPP (n = 4) and we measured V1 responsiveness again at 0 and 3 days. V1 responsiveness to contralateral eye stimulation as well as V1 activity elicited by visual stimulation of the ipsilateral eye did not change during the time tested (contra: 0 d vs 3 d: *p* = 0.25; ipsi: 0 d vs 3 d: *p* = 0.14; paired *t*-tests; Fig 9a and 9b; Table 2). Thus, the ODI remained unchanged in these mice (0 d vs 3 d: *p* = 0.21; paired *t*-tests; Fig 9c, Table 2). These data show that systemic administration of CPP blocks WD induced cross-modal plasticity in V1. Taken together, our results suggest that NMDA receptor activation is necessary to provoke cross-modal strengthening of sensory driven activity in a spared sensory cortex.

### WD cross-modally improves visual performance

As a next step we investigated whether the V1 response alterations, observed after WD or AD are also reflected at the level of visually mediated behavior. In a recent study we could already demonstrate that WD markedly refined V1 mediated visual performance as revealed by visual water task experiments [3]. Another example of visual behaviors is the so called optokinetic reflex (OKR), a head and eye movement, mediated by subcortical structures, which stabilizes images on the retina [47]. Interestingly, previous studies could show that V1 activity can modulate the OKR [27, 47]. We therefore hypothesized that the observed cross-modally induced changes of visually driven V1 activity (after WD or AD) might also lead to changes of the OKR. We therefore investigated the repercussions of WD and AD on spatial frequency and contrasts sensitivity of OKR using a virtual optomotor system [26].

**Fig 10:**
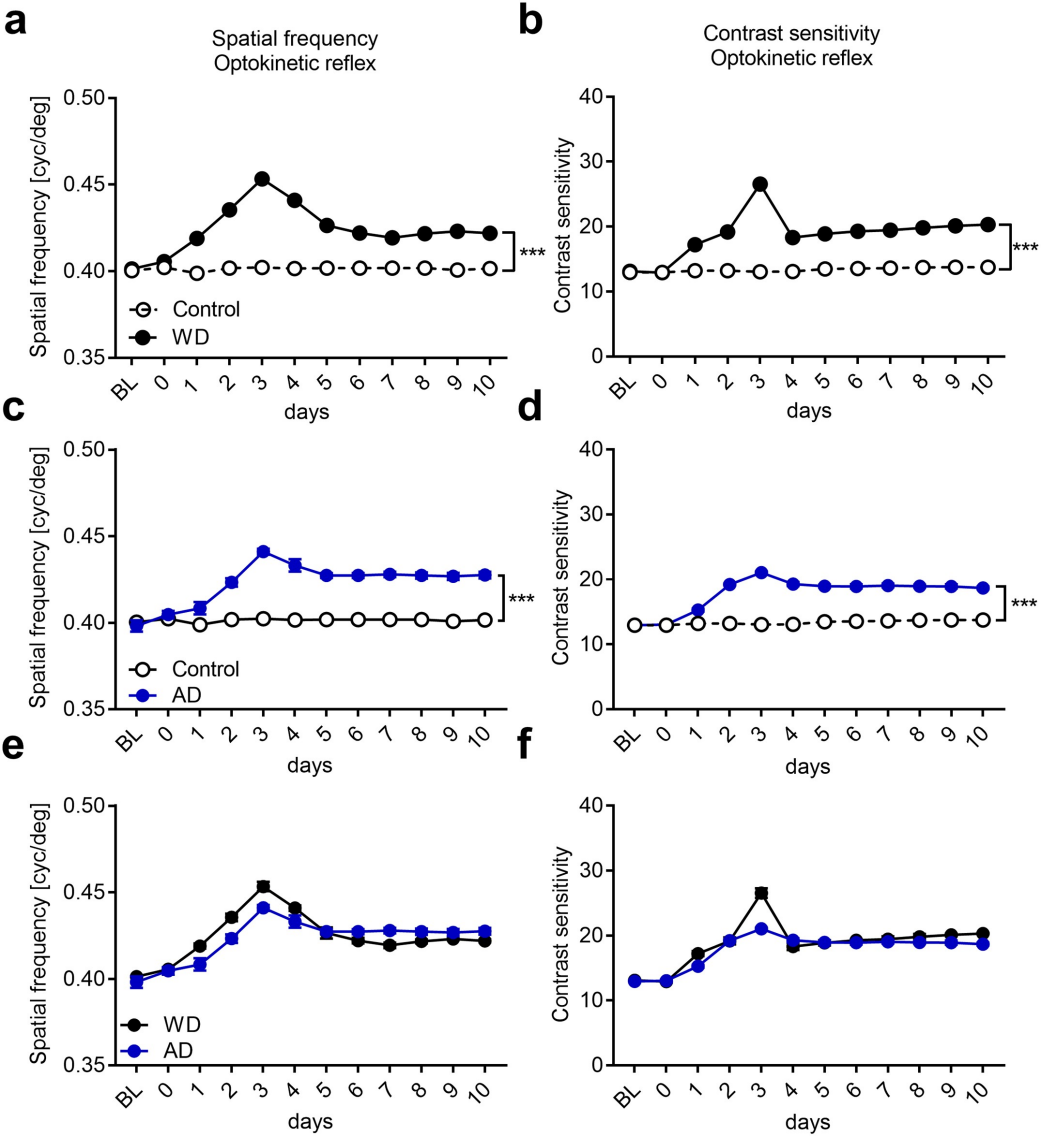
Both WD and AD cross-modally provoke a potentiation of the visual OKR. (**a**) In control mice (n = 4), spatial frequency thresholds did not change over the whole time period tested. However, after WD (n = 4) there was a marked improvement of the spatial frequency sensitivity which reached a peak on day 3. Subsequently, spatial frequency thresholds levels slightly decreased and remained at a stable level above control values until 10 days after WD. (**b**) Contrast sensitivity of the OKR in control mice remained unchanged over 10 days. After WD, contrast thresholds massively improved until day 3. After a slight decrease at 4 days after WD, values then remained at a stable level above control values until 10 days after WD. (**c**) We used values of the same control mice like in Fig 4a. AD (n = 4) led to a marked increase of spatial frequency thresholds peaking at 3 days. Subsequently, spatial frequency sensitivity slightly decreased until day 5 after AD and remained at a stable level above control values until 10 days after AD. (**d**) Contrast thresholds markedly increased until day 3 after AD which was followed by a slight decrease to a stable level above contrast values of control mice. (**e, f**) WD or AD led to similar improvements of the OKR. Open and filled circles represent mean values together with the s.e.m. However, vertical lines of s.e.m. are often occluded by data symbols. ***p<0.001.

First, we investigated the effects of WD on visual acuity. For this OKR thresholds obtained after visual stimulation of either the right or left eye were measured daily for a period of 10 days. Baseline values of WD mice (n = 4, Fig 10a and 10b) were always measured before WD, whereas values measured on day 0 represent measurements obtained 4–5 hours after the surgery for WD. Control mice (n = 4) remained untreated. Quantitative analysis using two-way ANOVA with repeated measurements revealed significant influences of group (F_1,6_ = 1165.94, *p*<0.0001) and time (F_11,66_ = 46.45, p<0.0001) and a significant interaction between the two (F_11,66_ = 46.45, p<0.0001). Post hoc analysis showed that reflex sensitivity for spatial frequency was unchanged in control animals (n = 4) over the whole time period tested. However, in the WD group (n = 4), there was a significant gradual enhancement of spatial frequency sensitivity of the OKR reaching a peak 3 days after WD, about 12% above control level (3 days: control vs WD: 0.40±0.001 (cpd(cycles per degree)) vs 0.45±0.0023 (cpd), *p*<0.0001; unpaired *t*-test followed by Bonferroni correction; Fig 10a). Spatial frequency thresholds levels then dropped down over the next two days, but persisted at a level about 5% above control values up to 10 days after WD (10 days: control vs WD: 0.40±0 (cpd) vs 0.42±0.00086 (cpd), *p*<0.0001; unpaired *t*-test followed by Bonferroni correction, Fig 10a). Contrast sensitivity of OKR was measured in the same control and WD mice at 0.2 cpd. Group (F_1,6_ = 656.48, p<0.0001) and time (F_11,66_ = 69.23, p<0.0001) had a significant influence on contrast thresholds and there was a significant interaction between both (F_11,66_ = 69.23, p<0.0001, tow-way ANOVA with repeated measurements). While contrast thresholds of control mice did not change over the whole time period tested, they gradually increased by almost 100% until day 3 after WD (3 days: control vs WD: 13.03±0.16 vs 26.55±0.74, *p*<0.0001, unpaired t-test followed by Bonferroni correction, Fig 10b) suggesting a substantial enhancement of contrast sensitivity due to WD. Subsequently, OKR contrast thresholds decreased again but then remained about 50% above control values between 4 and 10 days after WD (10 days: control vs WD: 13.73±0.26 vs 20.31±0.10; *p*<0.0001, unpaired t-test followed by Bonferroni correction; Fig 10b). Taken together, our results suggest that WD cross-modally improves behavioral OKR spatial frequency and contrast sensitivity.

As a next step, we examined whether also AD (n = 4) affects the visual OKR. Quantitative analysis using a two-way ANOVA with repeated measurements showed that group (F_1,6_ = 654.78, p<0.0001) and time (F_11,66_ = 24.3, p<0.0001) had a significant influence on spatial frequency thresholds. In addition, we found a significance interaction between group and time (F_11,66_ = 20.65, p<0.0001, two-way ANOVA with repeated measurements). As shown in Fig 10c there was a gradual increase of spatial frequency sensitivity until 3 days after AD (3 days: control vs AD: 0.40 ±0.001 (cpd) vs 0.44±0.002; *p*<0.0001; unpaired *t*-test followed by Bonferroni correction; Fig 10c) which was followed by a slight decrease over the next two days to a stable level above control values until day 10 after AD (10 days: control vs AD: 0.40±0 vs 0.43±0.002, *p* = 0.0002; unpaired *t*-test followed by Bonferroni correction; Fig 10c). Group (F_1,6_ = 317.79, p<0.0001) and time (F_11,66_ = 143.39, p<0.0001) also had a significant influence on contrast thresholds and there was a significant interaction between both (F_11,66_ = 112.92, p<0.0001, two-way ANOVA with repeated measurements). Contrast thresholds also significantly increased until 3 days after AD (3 days: control vs AD: 13.03±0.16 vs 12.06±0.16, *p*<0.0001, unpaired t-test followed by Bonferroni correction; Fig 10d) and then remained at a higher level above control measurements until day 10 (10 days: control vs AD: 13.73±0.26 vs 18.7±0.21, *p*<0.0001, unpaired t-test followed by Bonferroni correction; Fig 10d). These data indicate that AD can cross-modally improve OKR sensitivity, too. Notably, both spatial frequency and contrast sensitivity changes found after AD were similar to changes observed after WD (Fig 10e and 10f). Taken together, our results strongly suggest that the deprivation of a non-visual modality leads to marked improvements of subcortically mediated visual behavior.

### Cross-modally induced enhancements of the OKR are partially V1 dependent

So far, we described that both WD and AD lead to a potentiation of the OKR. Interestingly, the highest values of OKR thresholds of spatial frequency and contrast as well were obtained 3 days after WD or AD, and thus, exactly at the same time point when visually driven V1 reached its peak. These results suggest that V1 might be involved in mediating OKR potentiation. In order to address this issue we combined WD and bilateral V1 aspiration (WD+V1aspi, n = 3) and measured spatial frequency and contrast thresholds of the OKR over the following 10 days. In mice of the control group we only aspirated V1 bilaterally (V1aspi only, n = 3). Baseline values were always measured before V1 aspiration, measurements at day 0 were obtained 4–5 h after WD and V1 aspiration.

**Fig 11:**
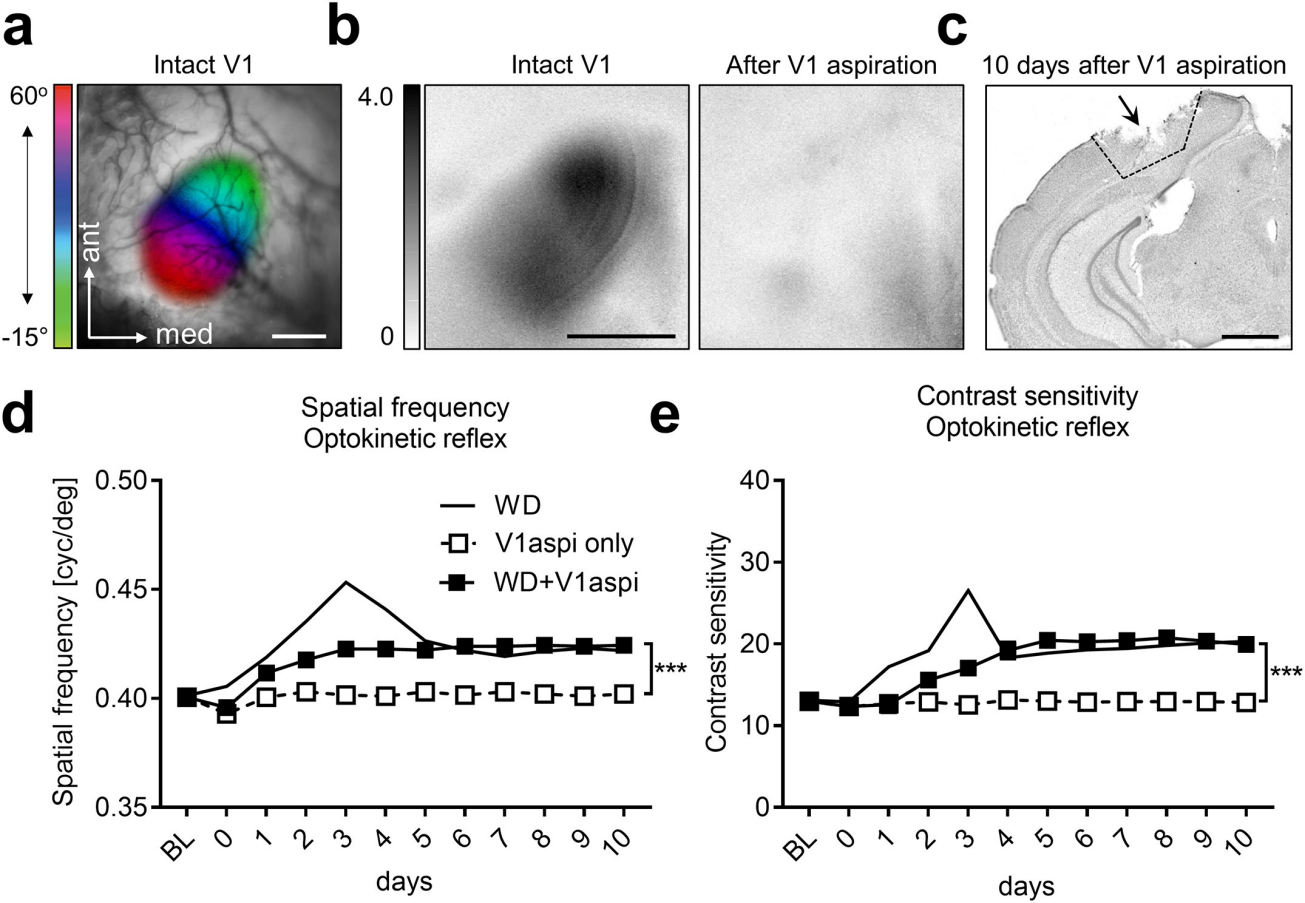
Aspiration of V1 reveals V1 contribution to cross-modally induced enhancements of the OKR. (**a**) V1 was located using intrinsic signal imaging. (**b**) Representative V1 amplitude maps elicited by visual stimulation before and after V1 aspiration. It is clearly visible that after V1 aspiration visually evoked cortical responses were absent demonstrating the efficiency of the aspiration surgery. (**c**) Nissl stained brain slice obtained 10 days after V1 aspiration. (**d**) Spatial frequency thresholds remained unchanged in mice after V1 aspiration only (n = 3). After combined WD and V1 aspiration (n = 3) spatial frequency sensitivity slightly increased until day 3 and remained at this level for the remaining 7 days. Interestingly, the strong peak of spatial frequency thresholds obtained 3 days after WD only (n = 4) was absent in mice after WD and V1 aspiration whereas the long-lasting improvement was almost identical in mice of both groups. (**e**) In WD only, V1aspi only and WD+V1aspi mice, the course of contrast sensitivity thresholds closely followed the course of spatial frequency thresholds in mice of the same groups. Taken together our data suggest that WD leads to V1 dependent and V1 independent improvements of the OKR. Open and filled squares represent mean values together with the s.e.m. However, vertical lines of s.e.m. are often occluded by data symbols.

For aspiration surgery we located the correct position of V1 using intrinsic signal imaging. Fig 11a depicts a representative visually evoked retinotopic polar map of V1, which was merged with a picture of the cortical blood vessel pattern of a normal mouse. Fig 11b (left) shows the corresponding amplitude map. We then aspirated V1, guided by blood vessel landmarks, through a small trepanation and performed a second optical imaging session to validate the efficiency of the surgery. As expected, after V1 aspiration visually evoked responses in the V1 area were completely abolished (Fig 11b, right, not quantified). These experiments confirm that the surgery for V1 aspiration was efficient and reliable since it completely abolished visually elicited V1 activity. Fig 11c shows a representative example of a brain slice 10 days after the aspiration of V1.

Quantitative analysis using a two-way ANOVA with repeated measurements showed that group (F_1,4_ = 77.27, p = 0.001) and time (F_11,44_ = 32.83, p<0.0001) had a significant influence on spatial frequency thresholds. In addition, there was a statistically significant interaction between group and time (F_11,44_ = 18.00, p<0.0001, two-way ANOVA with repeated measurements). As shown in Fig 11d the spatial frequency sensitivity of mice in that we only aspirated V1 (n = 3) remained almost unchanged for 10 days. However, if we combined WD and V1 aspiration, spatial frequency thresholds of the OKR slightly increased until day 3 and remained at this level for the whole time period tested (3 days: V1aspi only vs WD+V1aspi: 0.40±0.001 vs 0.42±0.001, 0.004; 10 days: 0.40±0.001 vs 0.42±0.001, *p* = 0.002; paired *t*-test followed by Bonferroni correction; Fig 11d). Interestingly, the peak of spatial frequency sensitivity at day 3, which we obtained in mice that only received a WD, was abolished in animals after concurrent WD and V1 aspiration. In contrast, the stable level of increased spatial frequency thresholds (between day 5 and 10) which was present in WD mice with combined V1 aspiration was practically identical to the increased stable level reached after WD only. Thus, these results suggest that the marked initial increase and decrease of spatial frequency sensitivity during the first 5 days after WD is mediated by V1. However, the long lasting improvement of spatial frequency thresholds after WD appeared to be V1 independent.

A similar result was obtained for contrast sensitivity of the OKR. Quantitative analysis using a two-way ANOVA with repeated measurements revealed significant influences of group (F_1,4_ = 44.05, p<0.003) and time (F_11,44_ = 37.08, p<0.0001) and a significant interaction between both (F_11,44_ = 31.83, p<0.0001). In mice in which we only aspirated V1 contrasts thresholds remained unchanged for 10 days (Fig 11e). When we combined WD and V1 aspiration, contrast thresholds gradually increased until day 5 and remained at this level for the following 5 days (5 days: control vs WD: 13.03±0.79 vs 20.43±0.54, *p* = 0.02; 10 days: 12.84±0.67 vs 19.94±0.29, *p* = 0.008; paired *t*-test followed by Bonferroni correction; Fig 11e). However, the peak of contrast sensitivity found in animals which only received a WD at day 3, was not present in mice after combined WD and V1 aspiration whereas the stable level of enhanced contrast thresholds (4–10 days) was almost identical in animals of both groups (Fig 11e). These data suggest that the transient strong improvement of contrast thresholds of the OKR found in mice after 3 days of WD alone is mediated by activity changes observed in V1. In contrast, the long lasting enhancement of contrast sensitivity does not require V1. Taken together, these results indicate that WD improves subcortically mediated visual spatial frequency and contrast sensitivity in both a V1 dependent and V1 independent manner.

### Cross-modally induced potentiation of OKR requires NMDA receptor activation

**Fig 12:**
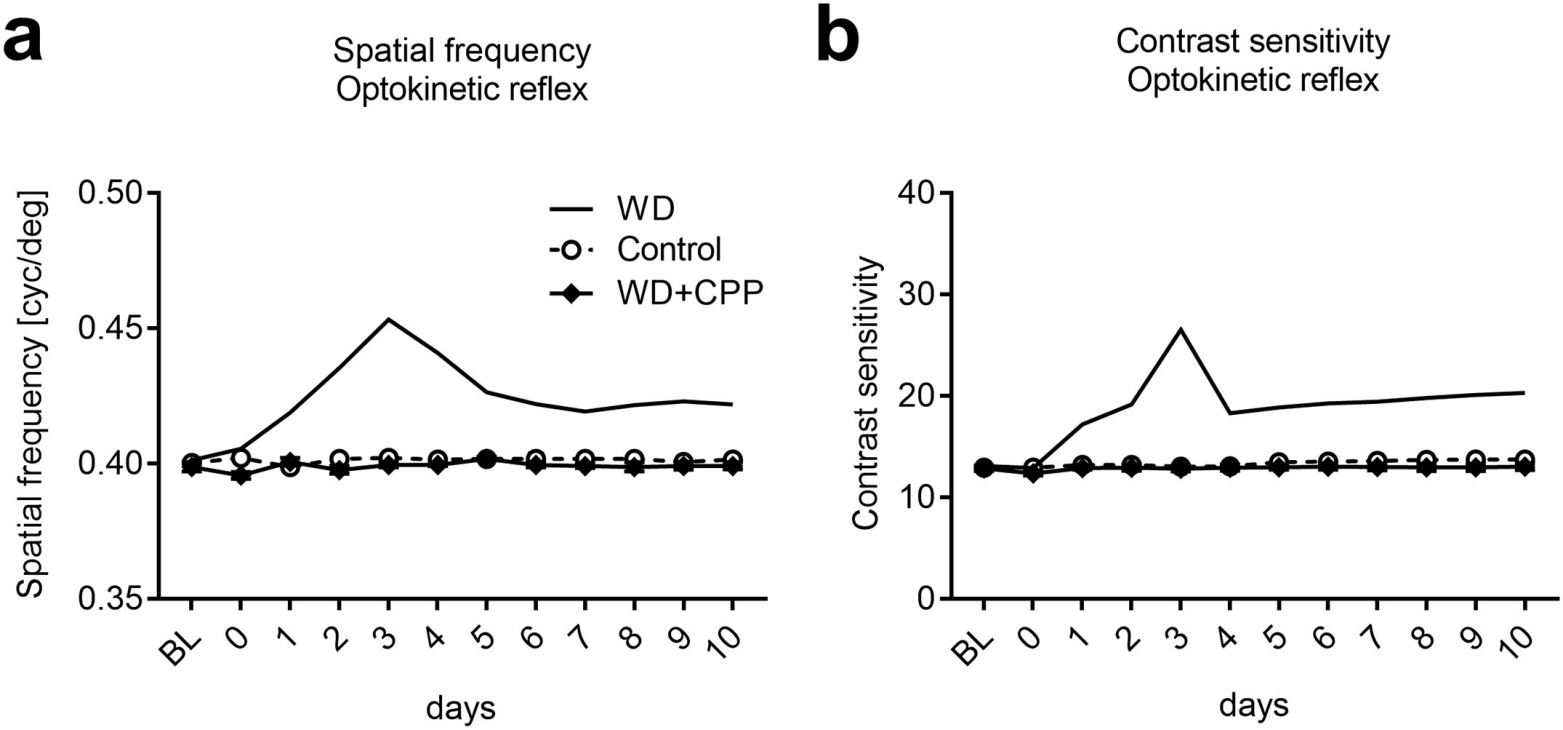
Cross-modally induced OKR potentiation requires NMDAR activation. (**a,b**) In both control mice (n = 4) and WD mice which received daily injections of CPP (n = 4) spatial frequency and contrast thresholds remained completely unchanged for 10 days. Hence, blocking NMDARs abolished both V1 dependent and V1 independent potentiation of OKR thresholds found after WD only.

We next examined whether NDMA receptors contribute to improvements of the OKR. For this, WD mice received daily CPP injections (WD+CPP; n = 4) and we measured spatial frequency and contrast thresholds again for 10 days. In these mice both spatial frequency and contrast sensitivity remained unchanged over the whole time period tested and were not different from values of untreated control animals (n = 4) (spatial frequency: group: F_1,6_ = 4.84, p = 0.07; time: F_11,66_ = 1.02, p = 0.44; interaction: F_11,66_ = 1.961, p = 0.149; contrast sensitivity: group: F_1,6_ = 1.63, p = 0.35; time: F_11,66_ = 6.8, p = 0.008; interaction: F_11,66_ = 2.097, p = 0.16; two-way ANOVA with repeated measurements; Fig 12a and 12b). Hence, CPP administrations abolished both the visual cortex dependent and independent improvements of the OKR thresholds observed in WD mice. These results suggest that potentiation of the OKR induced by the deprivation of a non-visual sense also depends on NMDAR activation, as shown above for the changes in V1 activity after WD.

## Discussion

In the present study we investigated the cross-modal effects of WD and AD on visually evoked V1 responses and visually mediated behavior in fully adult mice. Strikingly, we found that both WD and AD transiently shifted the OD in V1 towards the input through the ipsilateral eye. These changes required patterned vision through the ipsilateral eye and were accompanied by an increase of the E/I ratio in V1, suggesting a cross-modal restoration of V1 plasticity. Moreover, the observed changes in V1 activity partially mediated potentiation of the OKR, a visual behavior predominantly mediated by subcortical structures. These results indicate that the late-onset loss of a non-visual sensory modality dramatically alters neuronal processing at different stages of the visual pathway of adult mice, which in turn improves visually dependent behavior.

It has been demonstrated that prolonged monocular visual deprivation (MD, 5–7 days) in “young adult” mice around 60 days of age leads to a shift of the OD which is mediated by an increase of V1 activity elicited by open (ipsilateral) eye stimulation [24, 46, 48]. However, as the capacity of the brain to undergo experience dependent plastic changes massively declines with aging [12, 13], this type of cortical plasticity is completely absent in mice older than 110 days [11]. Strikingly, we show here that 3 days of WD or AD in mice of this age lead to V1 activity changes which resemble the type of OD plasticity in younger mice, since OD changes were also mediated by an increased V1 responsiveness to ipsilateral eye stimulation (Figs 3 **and** 4). Hence, our data provide evidence that WD or AD can rapidly restore V1 plasticity in fully adult mice. However, in contrast to the classical, now more than 50 years old paradigm showing that MD can induce alterations of OD [12, 14, 32], the cross-modally induced OD shifts described here took place without any visual deprivation. Thus, to the best of our knowledge, we demonstrate here for the first time that, at least in mice, OD in V1 can be altered by deprivations of non-visual sensory modalities, too.

Previous studies suggested that changes in the cortical E/I ratio in favor of excitation are the central hub for the restoration of cortical plasticity [49, 50]. For instance, dark exposure [39, 43], environmental enrichment [41, 44] and fluoxetine administrations [42], all cause lower inhibition and thus higher excitation levels in V1 and restore OD plasticity in V1 of fully adult mice. Moreover, these OD shifts can be prevented by artificially increasing cortical GABAergic inhibition by diazepam, a positive allosteric modulator of GABAA receptors [42–44], suggesting that a reduction of cortical inhibition is the common threat that re-induces cortical plasticity. We found that 3 days of WD or AD also raise the cortical E/I ratio in V1, in this case however, by increasing glutamate concentration in V1 (Fig 8). This was accompanied by a marked OD shift (Figs 3 **and** 4). These results are in accordance with previous studies demonstrating that treatments causing higher glutamate release in cortical synapses can reestablish cortical synaptic plasticity in adult mice [51, 52], suggesting that levels of the excitatory neurotransmitter glutamate also play an important role in regulating cortical plasticity. In addition, we found that increasing cortical inhibition by diazepam, and thus, re-decreasing the E/I ratio in V1, could abolish these OD shifts (Fig 9), indicating that the artificially increased GABAergic inhibition could compensate for increased glutamatergic excitation. Thus, our results suggest that increased cortical glutamate levels and thus, the increase of the E/I ratio in V1 was necessary for cross-modally induced OD changes. Hence, it can be assumed that deprivation of a non-visual sensory modality reinstates higher plasticity levels in V1, which then allow the visual input to re-shape V1 circuits. However, determining cortical GABA and glutamate levels by post mortem HPLC analysis of brain micro punches, as performed in the present study, is a relativity crude method to measure the cortical E/I ratio. This method cannot distinguish between intracellular GABA and the biologically active extracellular GABA in the synaptic cleft. Thus, to get a deeper mechanistically view into the cross-modally cortical alterations of the E/I balance, future studies could use the more precise *in vivo* micro dialysis to measure GABA and glutamate levels [41, 42, 45] or apply electrophysiological approaches [44]. However, as we found a general cross-modal increase of glutamate levels, it is likely that is, indeed, accompanied by increased cortical excitation.

There is increasing evidence that MD induced OD plasticity in young adult mice is mediated by long term potentiation (LTP)-like synaptic changes that lead to an increase of ipsilateral eye input to V1, as they require the NMDA receptor [24, 46, 48, 53]. In contrast, NMDAR dependent plasticity is also completely absent in fully adult mice older than 110 days [7, 46, 53–55]. However, here we show that the cross-modally induced OD shift after 3 days of WD or AD in mice of this age, also depends on NMDA receptors (Fig 9), suggesting that NMDAR function is reestablished in V1 after non-visual sensory deprivations. This conclusion is further supported by important recent studies: First, both WD and AD together with MD for 7 days can cross-modally restore NMDA dependent OD plasticity in fully adult mice [15, 56]. And second, AD has been shown to reactivate thalamocortical plasticity in V1, such as LTP, which was accompanied by a potentiated function of NMDAs in the adult V1 [57]. All these studies indicate a pivotal role of this particular receptor in mediating cross-modal plasticity in spared primary sensory cortices. However, as we administrated the NMDAR blocker CPP systemically, we cannot make statements on the precise location where this receptor is required to mediate cross-modal adaptations. It might be the visual cortex [24, 46, 57] but, as recent studies demonstrated that already neurons in the thalamus play a role in OD plasticity [58–60], it is possible that NMDARs, are already required in earlier structures of the visual pathway.

Previous studies have demonstrated that the deprivation of one sense for only a few days strengthens thalamo-cortical and layer 4 to 2/3 synapses in a spared primary sensory cortex [9, 10, 36]. In accordance with this finding, we here demonstrate that WD cross-modally increased the AMPAR mediated mEPSC amplitudes in layer 4 of V1, suggesting a strengthening of layer 4 synapses (Fig 7). At least a part of these strengthened synapses most likely represent thalamo-cortical synapses for several reasons: first, strengthening of thalamo-cortical synapses leads to increased sensory driven responsiveness of primary sensory cortices [10, 38]. This is in line with our imaging results, as evoked V1 activity was increased 3 d after WD. And second, visual input through the ipsilateral eye is required to mediate V1 activity alterations (Fig 6) suggesting an involvement of synapses and NMDARs on the visual pathway from the ipsilateral eye to V1. In summary, these results suggest that cross-modal plasticity, even in the adult spared sensory cortex is a form of experience dependent synaptic plasticity similar to long-term potentiation (LTP), as already demonstrated recently [6, 57]. Furthermore, our results demonstrate a high importance of V1 input through the ipsilateral eye for cross-modal plasticity in V1. However, further studies are required to examine the precise mechanisms underlying the surprising finding that exclusively the pathway of the ipsilateral eye to the binocular V1 seems to be affected by the deprivation of non-visual senses.

Previous studies reported that a prolonged sensory deprivation (for 7 days) leads to a decrease of AMPAR mediated mEPSC amplitudes in layers 2/3 of the spared primary sensory cortex [20, 61]. Hence, it was speculated that an initial strengthening of synapses in the remaining sensory cortices (after 2–3 days) is followed by a decrease in synaptic transmission after 7 days of sensory deprivation [9]. Our functional data of V1 responsiveness support this hypothesis as 7 days after WD or AD V1 responses were completely restored back to baseline levels (Figs 3 **and** 4). Thus, our data suggest that the restoration of normal OD levels is mediated by homeostatic mechanisms like synaptic down-scaling [20, 61] and/or cross-modally induced reduction of lateral input strength in layers 2/3 as shown previously [36].

Here we show that 3 days of either WD or AD alone induced a strengthening of V1 input through the ipsilateral eye, which was, however, followed by a recovery of normal V1 activity levels after 7 days (Figs 3 **and** 4). Recently, we also showed that 7 days of WD or AD alone did not lead to alterations of V1 activity [15]. However, when we combined WD or AD with MD of the contralateral eye for 7 days, responsiveness in V1 evoked by ipsilateral (open) eye stimulation was enhanced [15, 56]. Remarkably, in the present study, the same enhancement was observed 3 days after WD or AD alone (Fig 3), or 3 days of WD or AD combined with MD of the contralateral eye (Fig 6). These results strongly suggest that a prolonged MD for 7 days in WD or AD mice keeps V1 input through the ipsilateral eye at a higher level. In other words, under these MD conditions there is no recovery of V1 activity elicited by ipsilateral eye stimulation.

What might be a potential explanation for these differential changes in V1 responsiveness? As mentioned above, the downregulation of the overshooting V1 activity in WD or AD mice without MD is most likely mediated by homeostatic mechanisms, which readjust neuronal activity levels after perturbations [20, 36, 61, 62]. However, if WD or AD is combined with MD, activity levels in V1 are generally lower. Therefore, homeostatic mechanisms are not induced.

We recently showed that 7–12 days of WD dramatically improved V1 mediated visual acuity and contrast sensitivity, as measured by behavioral visual water task experiments [3]. Hence, the transient increase of V1 responsiveness 3 days after WD (or AD) described in the present study might be a necessary cortical alteration for later V1 dependent visual improvements that might compensate for the loss of somatosensation or audition, which in normal rodents provides essential information about their environment [7]. Here, we further demonstrate that both WD and AD also provoked a fast and a long-lasting improvement of the optokinetic reflex (OKR), another type of visual behavior mainly mediated by subcortical structures including the cerebellum and vestibular nuclei [47]. Previous studies demonstrated enhanced OKR sensitivity in rodents after MD [27], vestibular impairments [47, 63] or daily threshold testing from eye opening into adulthood [64]. Here, however, we provide the first evidence that OKR improvements can also be induced by depriving somatosensation or audition (Figs 10 **and** 11). We found that both, WD and AD resulted in three distinct phases of altered OKR sensitivity: During the first phase, OKR sensitivity markedly increased and peaked at 3 days after WD or. The second phase was characterized by a drop of OKR thresholds, lasting for 2–3 days. In phase three OKR thresholds stabilized and remained at a level above control values. Phase one and two appeared to be V1 dependent. These result are in line with previous studies demonstrating that V1 is involved in enhancements of OKR sensitivity [27, 47, 64]. Interestingly, the transient activity peak in V1 3 days after WD or AD temporally matched the peak of OKR improvements. These results suggest that cortico-fugal projections might transmit cross-modally induced V1 activity changes to subcortical structures which then act to mediate the compensatory potentiation of OKR. This hypothesis is supported by the findings that cortico-fugal projections can indeed modulate sensory induced behaviors [65, 66]. However, the third phase, where OKR sensitivity remained at a stable level above baseline for at least 10 days, was V1 independent as mice with removed whiskers (WD) and aspirated V1 also reached this enhanced level (Fig 11). However, we have to mention that V1 aspiration, as performed in the present study, is a relatively crude way to investigate the necessity of V1 in OKR changes, as also fibers of passage might be affected. Future studies should, hence, use more refined alternatives such as silencing V1 using muscimol or by optogenetical activation of inhibitory neurons in this region. However, as it has been shown that V1 aspiration and silencing V1 using muscimol affect the OKR in a similar manner [27], we believe that our approach, indeed, revealed a crucial role of V1 in mediating cross-modally induced OKR changes. Together with our previous finding that WD leads to an enhancement of visual performance in the visual water task [3], our OKR data indicate that the deprivation of non-visual senses provokes a general long-lasting compensatory improvement of visually mediated behaviors. Interestingly, like cross-modally provoked V1 activity changes, potentiation of the OKR could be completely abolished by antagonizing NMDARs (Fig 12). These results then suggest that NMDAR in different structures of the visual pathway are instrumental in the mediation of cross-modal effects.

In summary, we could demonstrate that the deprivation of non-visual sensory modalities transiently changes OD in V1. We postulate that reducing either somatosensory or auditory input cross-modally re-installs V1 plasticity in fully adult mice, allowing visual inputs to compensatorily re-shape V1 circuits. While further studies are needed to clarify the precise mechanisms underlying this novel and surprising finding, the present results already emphasize the power of cross-modal plasticity to re-open a window of high plasticity in the fully adult cortex far beyond any sensory critical period.
